# One-Step Preservation of Phosphoproteins and Tissue Morphology at Room Temperature for Diagnostic and Research Specimens

**DOI:** 10.1371/journal.pone.0023780

**Published:** 2011-08-17

**Authors:** Claudius Mueller, Kirsten H. Edmiston, Calvin Carpenter, Eoin Gaffney, Ciara Ryan, Ronan Ward, Susan White, Lorenzo Memeo, Cristina Colarossi, Emanuel F. Petricoin, Lance A. Liotta, Virginia Espina

**Affiliations:** 1 Center for Applied Proteomics and Molecular Medicine, George Mason University, Manassas, Virginia, United States of America; 2 Cancer Translational Research Center, Inova Fairfax Hospital, Inova Health System, Falls Church, Virginia, United States of America; 3 Center for Biodefense and Infectious Disease, George Mason University, Manassas, Virginia, United States of America; 4 St. James's Hospital, Dublin, Ireland; 5 Pathology Unit, Mediterranean Institute of Oncology, Catania, Italy; Food and Drug Administration, United States of America

## Abstract

**Background:**

There is an urgent need to measure phosphorylated cell signaling proteins in cancer tissue for the individualization of molecular targeted kinase inhibitor therapy. However, phosphoproteins fluctuate rapidly following tissue procurement. Snap-freezing preserves phosphoproteins, but is unavailable in most clinics and compromises diagnostic morphology. Formalin fixation preserves tissue histomorphology, but penetrates tissue slowly, and is unsuitable for stabilizing phosphoproteins. We originated and evaluated a novel one-step biomarker and histology preservative (BHP) chemistry that stabilizes signaling protein phosphorylation and retains formalin-like tissue histomorphology with equivalent immunohistochemistry in a *single* paraffin block.

**Results:**

Total protein yield extracted from BHP-fixed, routine paraffin-embedded mouse liver was 100% compared to snap-frozen tissue. The abundance of 14 phosphorylated proteins was found to be stable over extended fixation times in BHP fixed paraffin embedded human colon mucosa. Compared to matched snap-frozen tissue, 8 phosphoproteins were equally preserved in mouse liver, while AMPKβ1 Ser108 was slightly elevated after BHP fixation. More than 25 tissues from mouse, cat and human specimens were evaluated for preservation of histomorphology. Selected tissues were evaluated in a multi-site, independent pathology review. Tissue fixed with BHP showed equivalent preservation of cytoplasmic and membrane cytomorphology, with significantly better nuclear chromatin preservation by BHP compared to formalin. Immunohistochemical staining of 13 non-phosphorylated proteins, including estrogen receptor alpha, progesterone receptor, Ki-67 and Her2, was equal to or stronger in BHP compared to formalin. BHP demonstrated significantly improved immunohistochemical detection of phosphorylated proteins ERK Thr202/Tyr204, GSK3-α/β Ser21/Ser9, p38-MAPK Thr180/Tyr182, eIF4G Ser1108 and Acetyl-CoA Carboxylase Ser79.

**Conclusion:**

In a single paraffin block BHP preserved the phosphorylation state of several signaling proteins at a level comparable to snap-freezing, while maintaining the full diagnostic immunohistochemical and histomorphologic detail of formalin fixation. This new tissue fixative has the potential to greatly facilitate personalized medicine, biobanking, and phospho-proteomic research.

## Introduction

Protein kinase inhibitors constitute a large percentage of current lead compounds for molecular targeted cancer therapy [1]. Pre-clinical assessment of kinase inhibitors requires a comprehensive elucidation of their on-target and off-target effects in tissue, rather than cell lines, as well as an understanding of activated/phosphorylated signaling pathways in individual patient tumor specimens. Therefore, the ability to accurately quantify phosphorylated proteins represents an urgent pre-clinical as well as clinical need. Clinically, sub-populations of patients that may respond to such targeted kinase inhibitors need to be identified for individualization of therapy. Since the signaling pathways constituting the drug targets are composed of post-translationally modified proteins, this information cannot be directly obtained by RNA transcript profiling. Consequently, accurate quantitative measurement of the state of phosphoprotein cellular signaling pathways directly in human diagnostic tissue samples will be a critical driver for the future of molecular diagnostics [2].

### Phosphoproteins are reactive in living tissue

Excised tissue is alive, and phosphoprotein signatures change very rapidly during “cold ischemia time”, when cells experience the traumatic injury of excision and adapt to the absence of vascular perfusion, ischemia, hypoxia, acidosis, accumulation of cellular waste, absence of electrolytes, and temperature changes [3], [4]. In as little as 30 minutes post-excision, drastic changes occur in the protein signaling pathways of the biopsy tissue [5]. In response to wounding, cytokine release, vascular hypotensive stress, hypoxia, and metabolic acidosis, a large surge of stress-related, hypoxia-related, and wound repair-related signal pathway proteins and transcription factors are induced in the tissue [6]–[8]. Depending on the processing delay time *ex vivo*, and reactive stage, the activation/phosphorylation levels of cell signal proteins are then elevated or suppressed in a manner which does not represent the biomarker levels at the time of excision [5], [9]. In fact, with increasing sensitivity of analytical methodologies, an inherent danger is measuring cell adaptations to *ex vivo* stresses as opposed to measuring the *in vivo* state of cell signaling kinases. This critical problem of pre-analytical variability is receiving specific attention by the United States National Cancer Institute (NCI) through the Office of Biorepositories and Biospecimen Research (OBBR), which has found current tissue sample handling techniques to be a major roadblock to future quality research and personalized medicine [3].

### The need for phosphoprotein preservation in a paraffin block

Adequate preservation of phosphoproteins is required, if accurate information about the state of protein signaling architecture and drug target activation states at the time of procurement are to be known. Currently, the only preservation method routinely used to adequately preserve phosphoproteins is snap-freezing in liquid nitrogen. However, low temperature (liquid nitrogen or dry ice) freezing, shipping, and long-term storage is expensive and is not available in many clinics. Freezing compromises diagnostic pathology accuracy due to water crystal formation that can disrupt cell membrane structure and osmotic effects manifested as cell swelling/shrinkage (freezing artifacts). Subdividing tissue, in which some tissue portions are set aside for freezing and others are used for standard formalin fixation and histology, is not desirable in the clinical diagnostic standard of care due to tissue heterogeneity. Microscopic examination of the entire biopsy specimen is required for accurate diagnosis [10]. Therefore, several independent biopsies, that can be difficult and clinically impossible to procure, must be collected from a single patient. Consequently if tissue is removed for research, and not subjected to histopathologic examination, vital diagnostic information may be lost. The ideal solution would be for all diagnostic and molecular analysis to be done on the same paraffin block of tissue.

Although a number of laboratories are therefore using formalin-fixed, paraffin-embedded (FFPE) tissue for the evaluation of phosphoprotein levels [11]–[13], previous studies have indicated that the activation state of signaling proteins is not adequately preserved in FFPE tissue [14], [15]. This is not surprising, since the formalin fixation process is slow and requires several hours of fixation even for small core needle biopsies [16]. During this fixation time period, the living cells are reacting *ex vivo* to stress and rapidly change the underpinning protein-phosphorylation dependent signaling networks from the *in vivo* biology to a signaling architecture related to the *ex vivo* state.

**Figure 1:**
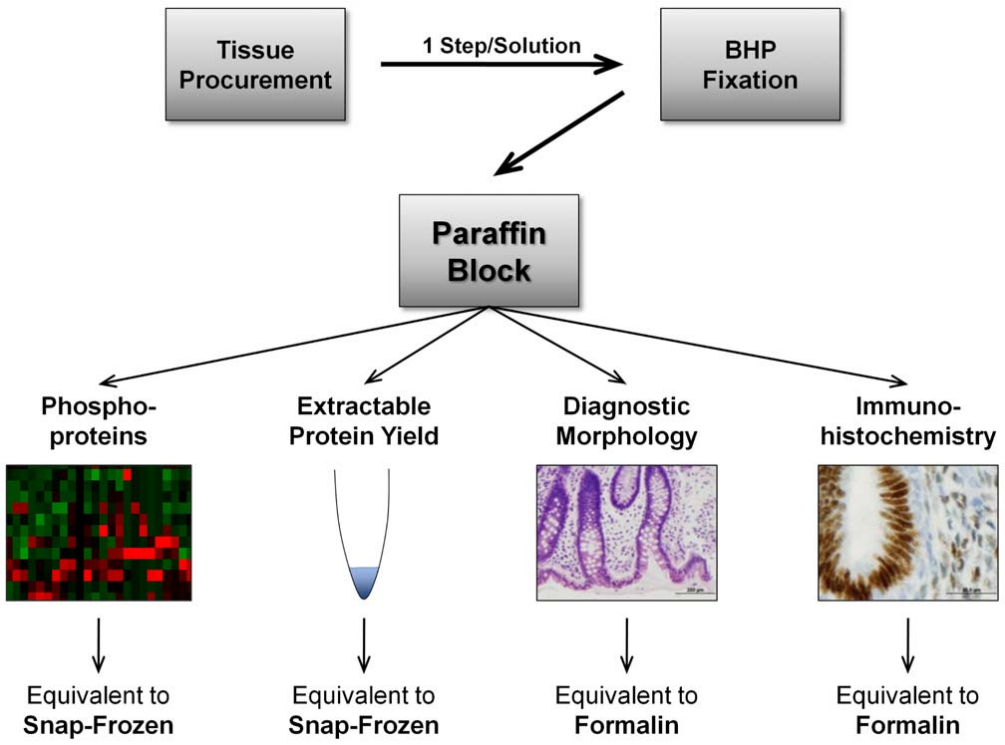
Workflow for one step, room-temperature phosphoprotein preservation with subsequent paraffin embedding. A novel tissue preservation solution, biomarker and tissue histology preservative (BHP), stabilizes phosphoproteins and cellular morphology at room temperature. The application combines the unique advantages of snap-freezing (immediate and excellent preservation of phosphoproteins, as well as high protein yield) and formalin fixation (preservation of tissue morphology and antigenicity) in one paraffin block.

The need for one-step room temperature preservation of phosphoproteins and histomorphology transcends diagnostic pathology to the broader research community. Preservation of phosphoproteins and tissue morphology within the *same* piece of tissue could have the potential to be adopted by clinical pathologists, biobanks, and open the way for parallel clinical diagnosis and molecular analysis. Based on this critical need, we asked the question if it is possible to stabilize phosphoproteins at room temperature in a paraffin block with diagnostic quality tissue morphology and preservation of protein antigenicity (Figure 1). To this end we designed a novel fixative chemistry based on the initially identified chemical principles required for an optimal phosphoprotein preservative [5]: (i) a combination of both kinase and phosphatase inhibitors to immediately arrest both sides of the kinase/phosphatase biochemical processes that occur continuously inside the cell and tissue, (ii) permeation enhancers to decrease penetration times into individual cells, (iii) a precipitating fixative with reversible cross-linkers to cross-link proteins beyond the active lifetime of the inhibitors, and (iv) an osmotically balanced buffer with a carboxylic acid to maintain tissue/cell morphology during fixation (Table 1). We previously demonstrated the successful application of this preservation strategy as a transport medium for downstream frozen tissue section applications [5]. However, preservation of phosphoproteins in frozen tissue is only useful in a research setting and is not applicable to routine medical practice or field-based research. Consequently we further developed the initial prototype phosphoprotein preservation chemistry as a solution compatible with room temperature collection followed by subsequent paraffin embedding. We evaluated the state of protein and post-translationally modified proteins from mouse, cat, and human tissues fixed in our biomarker and histology preservative (BHP) for (i) retention of phosphorylated levels over extended fixation time and in comparison to snap-frozen material, (iii) overall yield of extractable biomolecules, (iv) preservation of tissue morphology in multiple organs and tissues, and (v) preservation of key diagnostic immunohistochemistry antigens.

**Table 1:** Biomarker and Histology Preservative (BHP) composition.

Ingredient	Function
Kinase inhibitors	Inhibition of kinase activity
Phosphatase inhibitors	Inhibition of phosphatase activity
Permeation enhancers	Increased penetration rate into tissue/cells
Reversible crosslinkers	Easily cleavable cross-linkage of proteins beyond the lifetime of inhibitors
Water based buffer solution	Maintenance of tissue/cell osmotic balance and shape during fixation
Precipitating fixative	Inhibition of enzymatic activity
Carboxylic acid	Maintenance of tissue morphology

## Results

### Protein yield from BHP fixed paraffin embedded tissue

**Figure 2:**
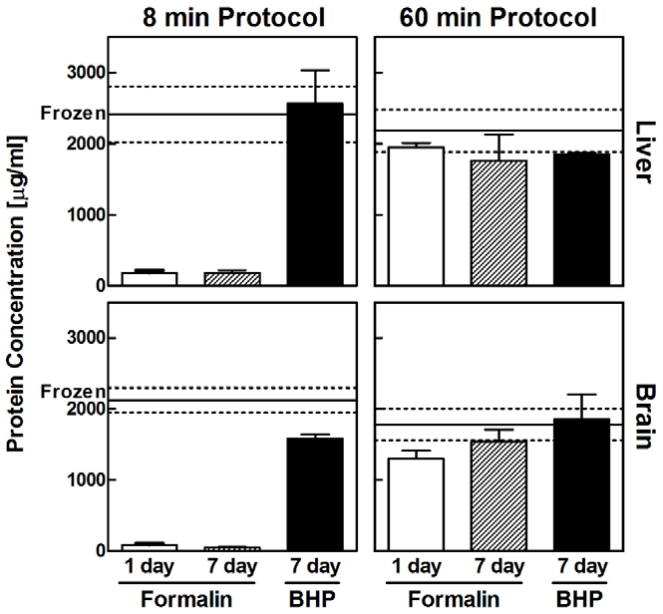
Protein yield from BHP-fixed tissue is equivalent to frozen tissue. Comparison of extractable protein yield from paraffin embedded BHP-fixed and formalin-fixed mouse brain and liver tissue to matched snap-frozen samples. Even after seven days of fixation in BHP and using a short eight-minute extraction protocol the total protein yield is very high compared to snap-frozen tissue. (white bar = formalin fixed 1 day ±SEM; gray bar = formalin fixed 7 days ±SEM; black bar = BHP fixed 7 days ±SEM).

The amount of protein that can be extracted from small tissue samples (i.e. core needle biopsies, laser capture microdissected material, etc.) is a key limiting factor for many proteomic analysis technologies. Adequately preserving tissue biomarkers at the expense of downstream protein recovery would therefore subvert the purpose of tissue fixation. We compared the total protein yield from BHP-preserved, paraffin-embedded tissue to matched samples that were either snap-frozen in liquid nitrogen or paraffin embedded after 10% neutral buffered formalin fixation (hereafter referred to as formalin) (Figure 2). Tissue sections were extracted in a volume of extraction buffer that maintained a standard ratio of tissue area to buffer volume. Tissue lysates were printed on reverse phase protein microarrays [17], [18]. Total protein was quantified on the reverse phase protein microarray by Sypro Ruby Protein Blot staining.

After the application of a standard eight minute extraction protocol, generally used for fresh frozen and/or microdissected tissue [19], the yield of protein per tissue area was 36-fold higher in mouse brain tissue fixed for seven days in BHP compared to matched samples fixed in formalin. Since the time of formalin fixation recommended by the American Society of Clinical Oncology/College of American Pathologists for typical applications such as the HercepTest is 6–48 hours [20] and to address the potential problem of overfixation, total protein was also extracted from mouse brain fixed in formalin for one day, which decreased the difference to 18-fold. Matched frozen brain tissue samples had a 1.3-fold higher protein yield than seven day fixed BHP samples and a 24-fold or 48-fold higher protein yield than one day or seven day fixed formalin samples (Figure 2).

The protein yield from mouse liver was 14-fold higher from tissue fixed for seven days in BHP compared to either one day or seven day fixation in formalin. No difference in protein yield was found between BHP fixed and frozen liver tissue.

To confirm if a higher extractability of protein from formalin fixed tissue, comparable to that obtained with the BHP fixed tissue, was possible using stronger extraction conditions, we slightly modified a 60 minute protocol reported by Ostasiewicz et al. [13]. While BHP-fixed brain and liver tissue retained high levels of extractable protein, the protein yield from one day and seven day fixed formalin tissue increased to levels comparable to that obtained by BHP fixed tissue (Figure 2, 60 minute protocol).

We were thus able to extract comparable levels of protein, using a short eight minute extraction protocol, from samples fixed for 7 days in BHP to matched snap-frozen tissue samples, supporting analysis with diagnostic or research specimens requiring short turn around times.

### Effect of BHP fixation time and tissue size on phosphoprotein preservation

**Figure 3:**
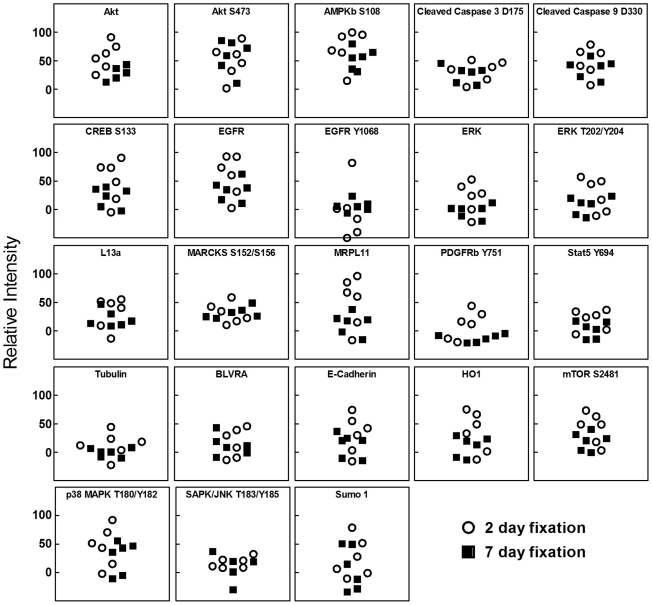
Phosphoprotein and total protein levels are stable for one week in BHP fixative. Six aliquots of human colon mucosa were either fixed for two or seven days in BHP and paraffin embedded. Reverse phase protein microarray analysis of 11 phosphoproteins, 10 total protein, and 2 cleaved protein modification endpoints revealed no significant difference in protein abundance due to fixation time (open circle = 2 day fixation, black square = 7 day fixation, Mann Whitney test, p>0.05).

In a busy clinical environment the time period between sample collection/start of tissue preservation (i.e. within the surgical operating suite) and the paraffin embedding process (i.e. within the pathological suite) cannot be controlled [3]. It is therefore critical that the fixation process, which can take place over several days, does not induce a selective shift in phosphoprotein abundance. We collected 12 samples of colon mucosa from a 52 year old male of which six were fixed for 48 hours in BHP, while the other six were fixed in BHP for seven days, followed by paraffin embedding. We then compared the level of 11 phosphorylated, 2 cleaved, and 10 non-phosphorylated proteins in all 12 samples (Figure 3). No statistically significant difference (p>0.05) was found between fixation times for any of the 23 proteins evaluated.

As recommended by clinical pathology guidelines [21], all samples were cut into pieces of less than 1 cm^3^ prior to fixation and processing. To address the question whether differences in tissue size would affect phosphoprotein preservation we correlated the level of 11 phosphoproteins with the respective tissue size (µm^2^, as measured by the Arcturus^XT^ platform) in all 12 samples of human colon mucosa described above. Spearman's rho analysis showed no correlation between tissue size and protein preservation (p>0.05) (Figure S1 and Table S1), indicating that phosphoprotein levels are independent of BHP fixation time and minor variations in tissue size. Since all 12 samples originated from a single patient, collection and processing times were equal between samples.

### Comparison of phosphoprotein levels between patients after BHP fixation

**Figure 4:**
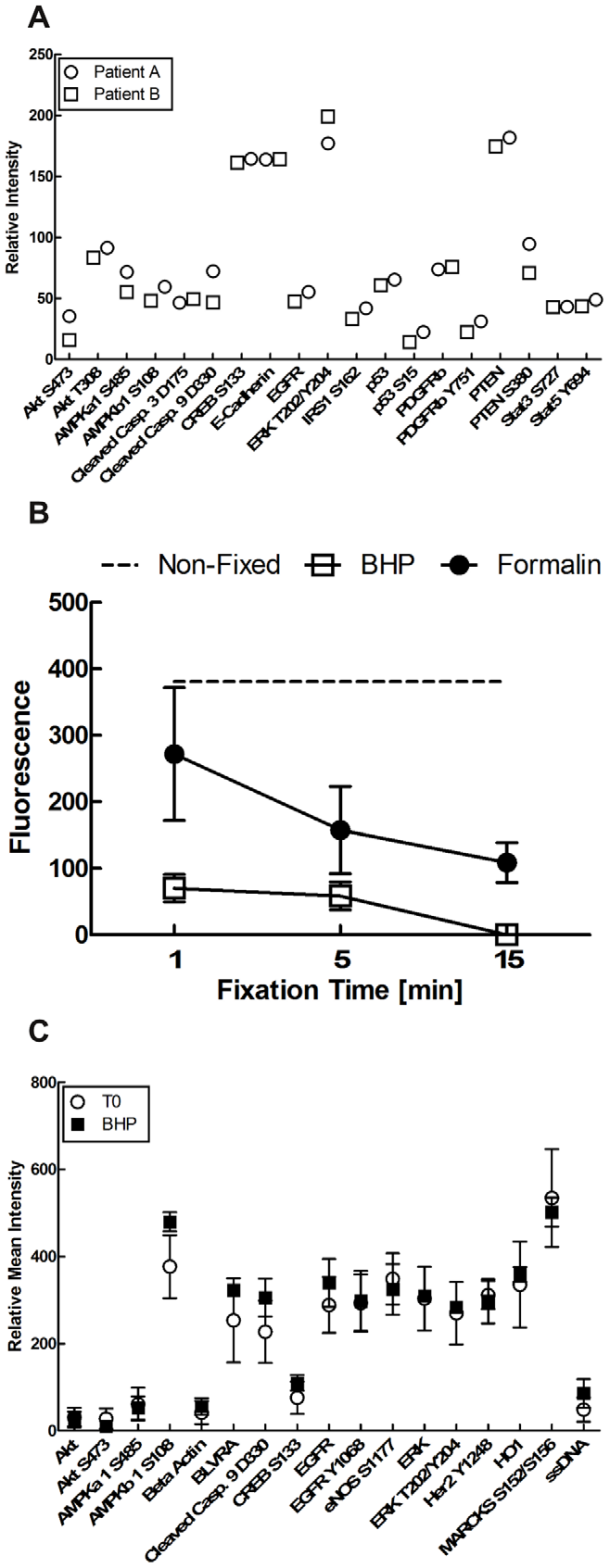
Evaluation of protein and phosphoprotein preservation by BHP fixation. (A) Slight tissue-architectural differences between patients don't appear to affect fixation efficiency by BHP. The reverse phase protein microarray analysis of 19 endpoints in human colon mucosa from two different patients revealed an almost identical pattern of post-translationally modified and total protein abundances. (B) BHP fixation rapidly reduces tissue tyrosine phosphatase activity. Human breast tissue samples were directly lysed or fixed in BHP or formalin for 1, 5 and 15 minutes prior to lysis. Residual total tyrosine phosphatase activity was measured using an ELISA. After one minute of BHP fixation phosphatase activity was reduced to 18% of non-fixed levels and was completely absent by 15 minutes of fixation. In contrast, formalin fixation retained 71% phosphatase activity after 1 minute of fixation and 29% by 15 minutes of fixation. (n = 2, black circle-formalin; white square = BHP, dashed line non-fixed; ±SEM). (C) BHP fixation followed by paraffin embedding yields comparable levels of post-translationally modified and total protein in mouse liver tissue compared to snap-frozen samples for 16/17 endpoints. Mouse liver (n = 3, ±SEM) was either paraffin embedded after fixing in BHP for seven days or snap-frozen immediately and analyzed on a reverse phase protein microarray (open circle = snap frozen; black square = BHP 7 day fixation).

We extended our analysis of phosphoprotein preservation between samples from different patients, where cellular composition, collection and processing times may not be exactly the same. Slight architectural and molecular differences in the same tissue from different patients could potentially result in variations in fixative penetration rate and kinase/phosphatase inhibition, which could lead to preservative induced alterations of phosphoprotein levels. Using reverse phase protein microarrays [17], [22], we compared the abundance of 19 phosphorylated and non-phosphorylated proteins in non-diseased human colon mucosa from two patients with a history of colon polyps (patient A: female age 45, and patient B: male age 56) (Figure 4A). Collection and processing times were similar for both samples. Patient A tissue was received 8 minutes after surgical excision. Tissue was immersed in BHP fixative 16 minutes after receipt, for a total elapsed time from collection of 24 minutes. Patient B tissue was received 9 minutes after surgical excision. Tissue was immersed in BHP fixative 14 minutes after receipt, for a total elapsed time from collection of 23 minutes. Tissue was fixed for 24 hours prior to paraffin embedding using an automated tissue processor with a standard ethanol protocol. The pattern of protein abundance between both patient samples was very similar and well within the variation expected between two individuals, supporting equivalent preservation of both samples.

### Effect of BHP fixation on the phosphorylation of site-specific residues on EGF receptor post EGF stimulation

**Figure 5:**
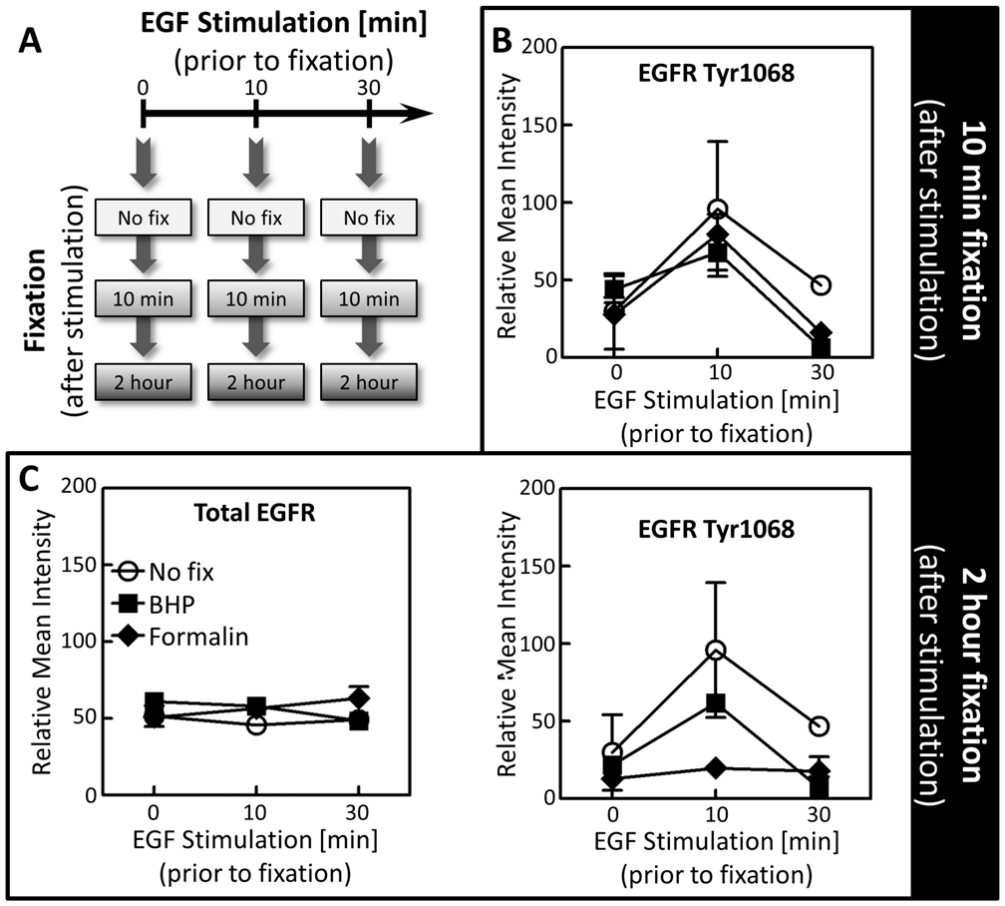
BHP fixation preserves dynamic changes in phospho-EGFR Tyr1068 abundance post stimulation. (A) Schematic of experimental setup. U266 cells were continuously stimulated with EGF for 30 minutes. Cell aliquots at 0, 10 and 30 minutes were removed and directly lysed or fixed in BHP or formalin prior to lysis. (B) After 10 minutes of fixation, BHP-fixed and formalin-fixed cells correctly maintain the expected pattern of phospho-EGFR Tyr1068 increase and decrease that is observed in non-fixed cells. (C) While BHP-fixed cells still maintain the expected phosphorylation pattern after two hours of fixation, this pattern is lost in formalin-fixed cells. (n = 3, black diamond = formalin; black square = BHP; open circle = non-fixed; ±SEM; error bars for the samples directly lysed after 30 minutes of EGF stimulation are very small and masked by the open circle symbol).

The minute by minute state of active phosphoprotein signaling pathways is a function of the dynamic and highly reactive phosphorylation and de-phosphorylation of proteins in response to external and internal stimuli. This fluctuating network needs to be promptly and permanently frozen in time to allow meaningful evaluation of cell signaling states. To evaluate the room temperature preservation of dynamic protein phosphorylation events, we stimulated U266 cells with epidermal growth factor (EGF) for up to 30 minutes. EGF stimulation is expected to lead to an increase in EGF receptor (EGFR) phosphorylation within the first 10 minutes [18], followed by an attenuation of phosphorylated receptor levels due to dephosphorylation, internalization, and degradation [23], [24]. At 0, 10 and 30 minute time points post stimulation cell aliquots were removed and lysed immediately or fixed with either formalin or BHP for either 10 minutes or for two hours prior to cell lysis (Figure 5A). Phosphoprotein levels were quantified by reverse phase protein microarray [17], [22]. As expected, EGFR phosphorylation at tyrosine residue 1068 (EGFR Tyr1068) increased after 10 minutes of stimulation with EGF, while decreasing by 30 minutes after continuous EGF exposure (Figure 5) [18]. The abundance of total EGFR (phosphorylated and non-phosphorylated) did not change during treatment (Figure 5C). Low levels of phospho-EGFR Tyr1068 at 0 minutes, increased phospho-EGFR Tyr1068 after 10 minutes and decreased phospho-EGFR Tyr1068 were also seen after BHP fixation. This exactly mirrored the pattern observed when cell aliquots were not fixed after stimulation, indicating stable preservation of dynamic phosphorylation processes by BHP. In contrast, formalin fixation of cell aliquots after stimulation induced a significant loss of phosphorylation, with low levels of phospho-EGFR Tyr1068 at the 10 minute stimulation time point. Similar protein concentrations of frozen, BHP fixed and formalin fixed cell lysates were printed on the array, therefore, the reduced signal intensity was not an artifact due to differences in protein quantity. Reducing the formalin-fixation time from two hours to ten minutes reestablished the originally observed phosphorylation pattern of EGFR Tyr1068 (Figure 5), further indicating that it is in fact the prolonged fixation by formalin that alters phospho-EGFR Tyr1068 levels. This confirms that BHP fixation is capable of capturing the dynamic phosphorylation pattern present in the cell at the time of fixation, while formalin fixation may not preserve this information adequately due to variations in fixation times.

### Effect of BHP fixation on tyrosine phosphatase activity

One possible explanation for the difference in phosphoprotein preservation by BHP and formalin was the retention of residual phosphatase activity during formalin fixation. During the fixation process phosphatase activity could lead to altered phosphorylation levels that do not accurately mirror pre-fixed phosphoprotein abundance. Using an ELISA we measured the total tyrosine phosphatase activity in pieces of cultured breast tissue after 1, 5, and 15 minutes of fixation with either BHP or formalin. One minute of BHP fixation reduced the total tyrosine phosphatase activity to 18% of non-fixed levels, while formalin fixed tissue extracts retained 71% phosphatase activity (Figure 4B). After 15 minutes of fixation BHP preserved tissue showed complete absence of tyrosine phosphatase activity, while phosphatase activity in formalin fixed tissue was still at 29% of non-fixed levels. It is therefore conceivable that residual phosphatase activity in formalin fixed samples could lead to falsely decreased phosphoprotein levels during tissue fixation (Figure 5).

### Comparison of phosphoprotein levels between BHP fixed and snap-frozen samples

Freezing is generally regarded as the standard protocol of protein preservation. To evaluate room temperature preservation of phosphoproteins in a paraffin block after BHP fixation, we measured the relative abundance of nine phosphorylated proteins, six non-phosphorylated proteins, one cleaved protein and single stranded DNA in mouse liver (strain ICR/CDI) using reverse phase protein microarrays (Figure 4C). Liver was selected for analysis due to the relative cellular homogeneity of this organ, thus minimizing any potential analytical artifacts due to tissue heterogeneity. No significant difference was found for any of the analytes. The exception was phosphorylated AMP-activated protein kinase (AMPK) β1 Ser108, where slightly higher levels were found after BHP fixation (Figure 4C).

One of the caveats of comparing snap-frozen tissue to fixed and paraffin embedded tissue is the retention of blood and/or other body fluid contaminants within the frozen tissue. These contaminants are largely washed away during the fixation and paraffin embedding process. To determine the impact of residual blood on the results of the frozen material, we also measured the abundance of single stranded DNA (ssDNA). Since erythrocytes don't possess DNA, significant blood contamination would decrease the ratio of ssDNA versus total protein amount. We observed comparable ratios of ssDNA/total protein in fixed and frozen samples, which indicates that a major impact on the data obtained from frozen material by residual blood contamination is unlikely.

The almost identical phosphorylation pattern of snap-frozen and BHP-fixed, paraffin-embedded tissue across nine phosphoproteins (Figure 4C) supports the original hypothesis that room-temperature preservation of phosphoproteins in a paraffin block is possible.

### Histology of paraffin embedded tissue fixed with BHP

**Figure 6:**
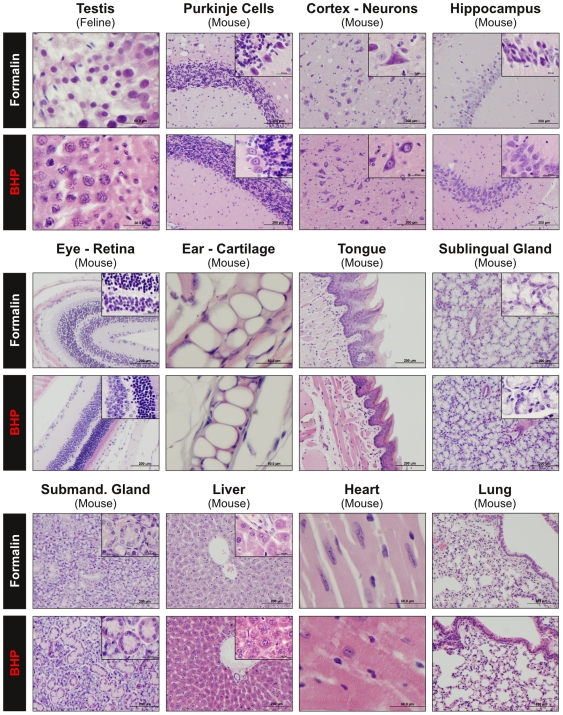
Comparison of tissue morphology between paraffin blocks of BHP-fixed or formalin-fixed mouse and feline tissue. BHP fixed or fomalin fixed matched tissue sections were stained with Hematoxylin and eosin (H&E). BHP fixation demonstrates equivalent preservation of cellular morphology with full cytoplasmic and membrane detail compared to formalin fixation. Nuclear structures (chromatin and nucleoli) are generally better preserved by BHP (20x magnification, inset 100x) (Continued in Figure 7) (For description of histological details see Text S1).

**Figure 7:**
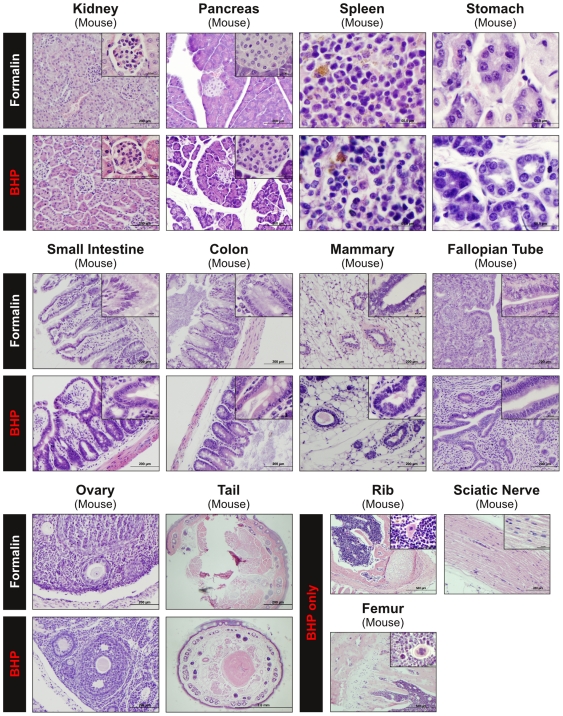
Comparison of tissue morphology between paraffin blocks of BHP-fixed or formalin-fixed mouse tissue. In addition to excellent preservation of cytoplasmic, membrane and nuclear detail, BHP-fixation eliminates the need for decalcification of bony tissue. Mouse tail, rib, and femur samples were fixed in BHP and paraffin embedded without decalcification prior to embedding. Bony structures remain intact and were able to be cut into sections (5 µm) using standard sectioning protocols (H&E stain, 20x magnification, inset 100x) (For description of histological details see Text S1).

The overarching goal of this study was the development of a new tissue fixative that provided both room-temperature preservation of phosphoproteins and diagnostic tissue histomorphology within the *same* paraffin block. Since tissue structure, complexity, and cellular composition vary greatly between tissue types we assessed the tissue and cellular morphology after BHP fixation and paraffin embedding in over 25 different mouse, cat and human tissues (Figures 6 and 7, Table S4, Text S1). Mouse tissues were selected for comparison because the time of collection and processing could be controlled for a comparative study whereas human biopsy specimens may exhibit wide variability in collection/processing protocols. Formalin fixed paraffin embedded matched tissues were selected for comparison because a) formalin is routinely used in diagnostic pathology, and b) a majority of archived tissue samples consist of FFPE tissues.

**Figure 8:**
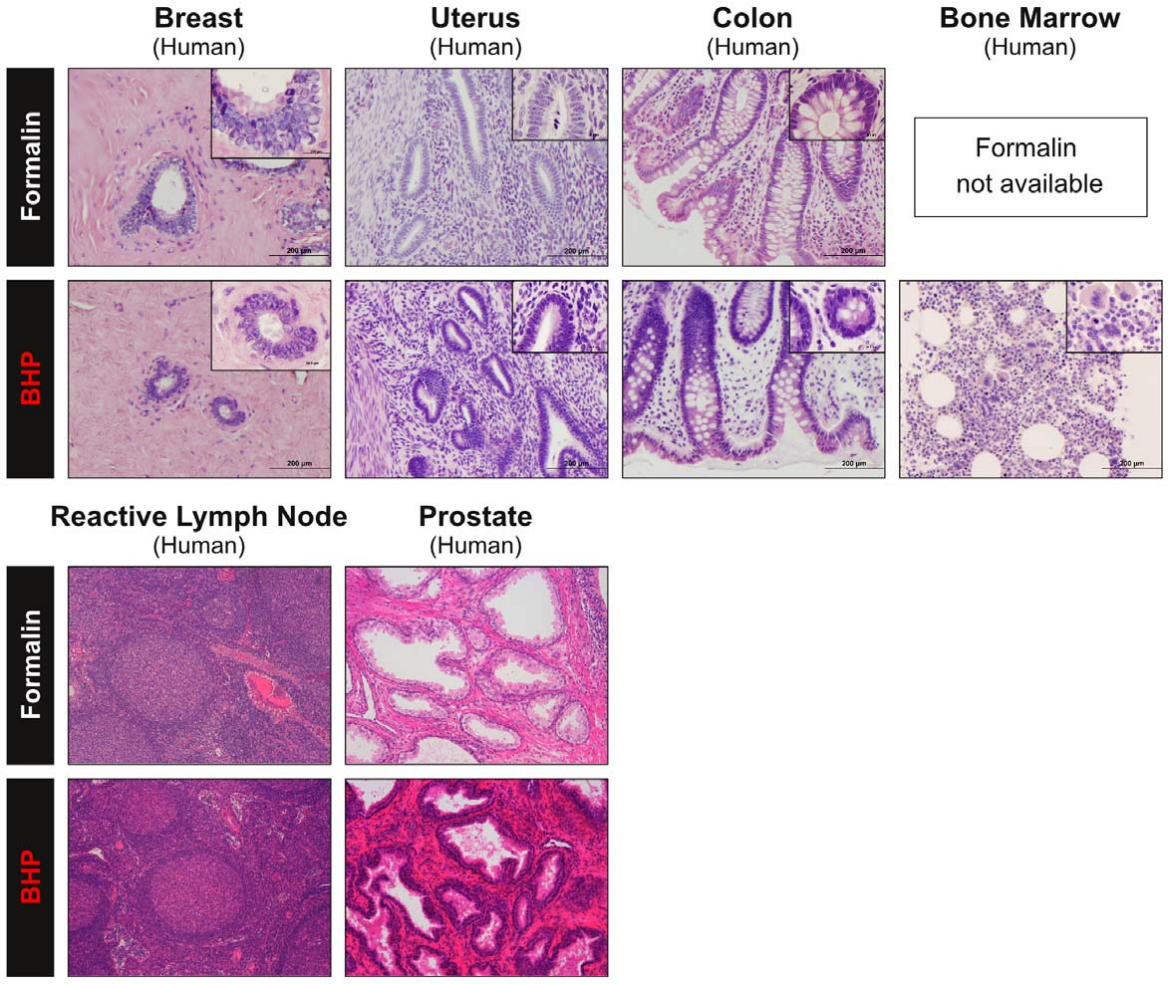
Human tissue fixed in BHP retains morphology equivalent to formalin. Matched human tissue samples were fixed in either BHP or formalin and paraffin embedded. The same pattern as seen for mouse and feline tissues (see Figures 5 and 6) were observed for human tissue fixed in the one-step biomarker and histology preservative: equivalent preservation of cytoplasmic and membrane detail, with superior preservation of nuclear structures as compared to matched formalin fixed paraffin embedded tissue (H&E stain, 20x magnification, inset 100x) (For description of histological details see Text S1).

In general, comparison with matched FFPE tissue showed BHP fixation achieved equivalent preservation of parenchymal, stromal, epithelial, and lymphoid histomorphology with full preservation of cell volume, cytoplasmic and membrane detail. Nuclear structures (nuclear membrane, chromatin and nucleoli) were better preserved by BHP. This was especially striking in feline testes, where chromatin strands were clearly visible after BHP fixation, whereas nuclei in formalin fixed testes did not present any chromatin detail (Figure 6, Text S1). Superior BHP preservation of nuclear structures and size was seen in all mouse tissues observed and all eight human tissue samples (Figure 6, 7, 8, Table 2, Text S1).

**Table 2:** Independent evaluation of human colon mucosa by two pathologists.

Criteria	Worse than FFPE	Equal to FFPE	Better than FFPE
Overall color fidelity		x	
Cell size		x	
Preservation of nuclear membrane		x	
Preservation of nucleoli			x
Preservation of overall cell structure		x	
Nuclear:Cytoplasmic ratio maintained		x	

BHP-fixed, paraffin-embedded tissue compared to formalin-fixed, paraffin-embedded (FFPE) tissue.

### BHP fixation obviates the need for decalcification of bony tissues

Bony tissue presents a special challenge for molecular profiling studies, since it cannot be cut into sections without being subjected to harsh decalcifying conditions. Several methods of decalcification have been shown to impair DNA and RNA recovery and reduce antigenicity for certain antigens [25]–[28]. BHP fixation completely eliminated the need for decalcification in 3×5 mm or smaller tissue samples. Human bone marrow core biopsies as well as mouse tail, rib, and femur were fixed in BHP, paraffin-embedded and sectioned with no limitations. Internal bone structures were well preserved, including growth plates in mouse femur and rib, as well as bone marrow trabeculae in human bone marrow cores (Figures 7 and 8, Text S1). Equally treated formalin-fixed bony tissue could not be sectioned properly, as shown in H&E stained sections of formalin-fixed mouse tail (Figure 7, Text S1).

### Effect of BHP fixation on nuclear size

**Figure 9:**
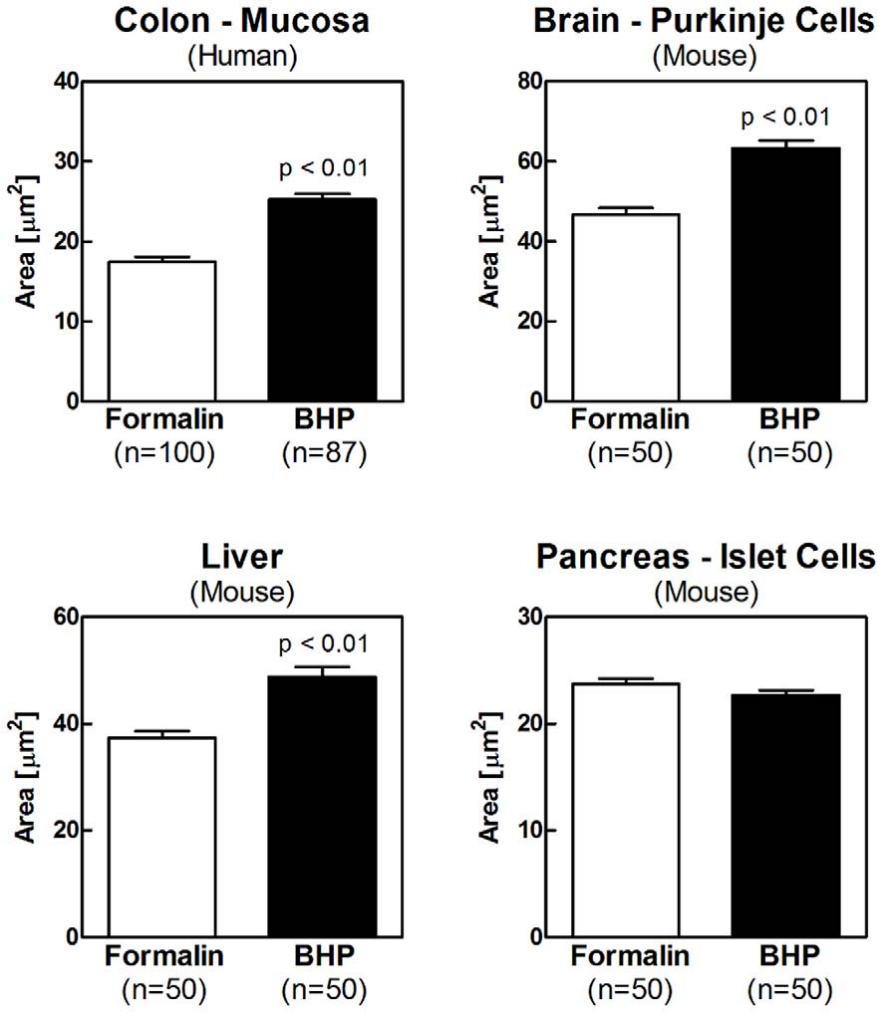
Nuclear size is maintained in tissue fixed with the novel biomarker and histology preservative. The nuclear area of BHP-fixed or formalin-fixed and paraffin-embedded human colon mucosa, mouse brain purkinje cells, mouse liver hepatocytes and mouse pancrease islet of Langerhans cells was measured using the Arcturus XT platform (n = 50–100). No shrinkage in nuclear size was observed after BHP fixation. (white bar = formalin; black bar = formalin ±SEM).

A common phenomenon of ethanol-based fixatives is the shrinkage of nuclei and cell volume after fixation [29]. Nuclei shrinkage and loss of nuclear detail can severely compromise diagnostic accuracy. Since BHP is a formalin-free fixation chemistry that contains ethanol we measured the size of 50–100 nuclei in four different tissues to assess the effect of alcohol on nuclear size and detail. After BHP fixation and paraffin embedding, the average size of nuclei from human colon mucosal epithelium cells, mouse brain purkinje neurons, and mouse liver hepatocytes was fully maintained and slightly increased compared to FFPE tissue. Mouse pancreas islet of Langerhans cells were equal in size in the two fixation types (Figure 9).

### Effect of BHP fixation on protein immunoreactivity

Immunohistochemical membrane, cyotplasmic and nuclear protein antigenicity is a critical component of clinical diagnosis. Without the preservation of antigenicity, along with tissue morphology and phosphoprotein abundance, in the *same* paraffin block, BHP fixation could not be applied to clinical diagnosis and would not be accepted by clinical pathologists. To show that our new fixation chemistry maintains antigenicity in human tissues for common diagnostic antigens, we selected 14 non-phosphorylated proteins, for analysis in an international, multi-center diagnostic anatomic pathology laboratory study. Proteins representing different sub-cellular locations were selected to show the preservation of specific cellular constituents (Table S6). We also evaluated Periodic Acid Schiff (PAS) and diastase-resistant PAS stain as polysaccharide/mucin specific stains, to demonstrate the compatibility of BHP fixed, paraffin embedded tissue with routine immunohistochemical protocols. Although staining and embedding protocols varied between institutions and for different antigens, each institution used their specific protocol to stain the matched formalin and BHP fixed tissues at the same time.

**Figure 10:**
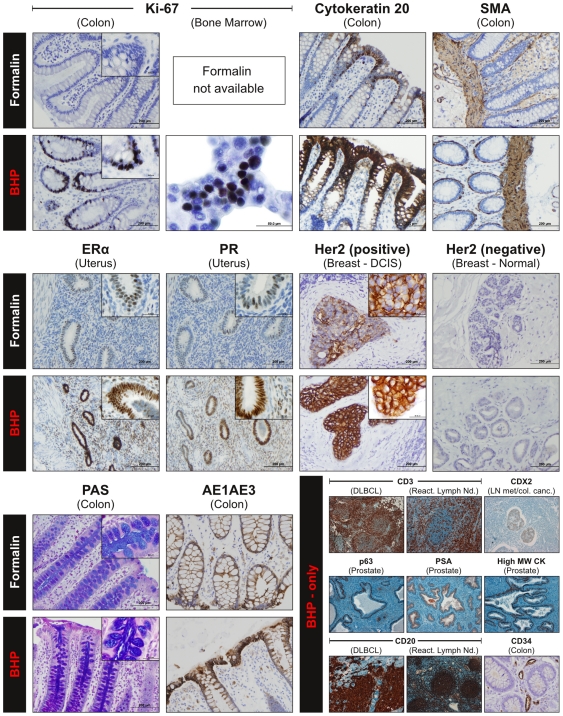
Biomarker and histology preservative shows equivalent or enhanced antigenicity by immunohistochemistry compared to FFPE. To show that our new fixation chemistries maintains antigenicity for common diagnostic antigens, the effect of BHP fixation on non-phosphorylated protein antigenicity was compared in human tissue fixed in BHP or formalin and paraffin embedded in an international, multi-center study. At each institution, sections were stained side-by-side, using the same protocol for each fixative. Staining and embedding protocols varied between institutions and for different antigens. Antigenicity was stronger for most endpoints in BHP-fixed tissue, with the exception of CDX2 (SMA  =  smooth muscle actin, ERα  =  estrogen receptor alpha, PR  =  progesterone receptor, PAS  =  Periodic Acid Schiff stain, DCIS  =  ductal carcinoma in-situ, DLBCL  =  diffuse large B-cell lymphoma, LN met/col. canc.  =  lymph node metastasis from colon cancer) (For description of histological details see Text S1).

**Figure 11:**
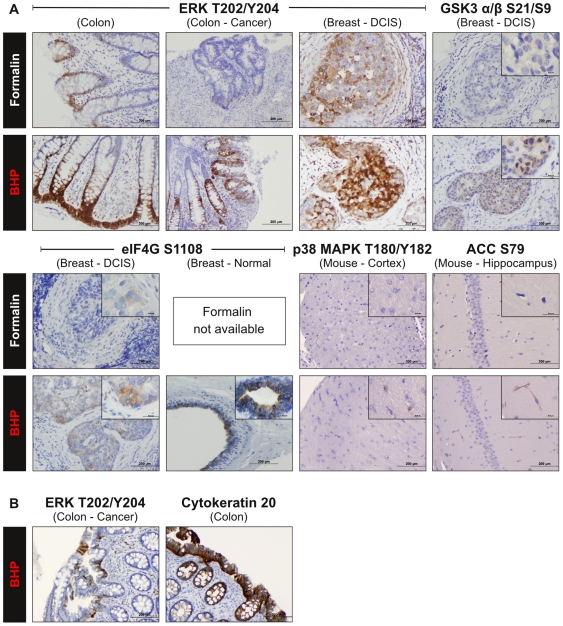
BHP fixed tissues retain phosphoprotein antigenicity with immunhistochemistry. (A) Effect of BHP fixation on phosphorylated protein antigenicity. Human and mouse tissues were fixed with BHP or formalin, followed by paraffin embedding. Sections were stained side-by-side, using the same protocol for each fixative. Staining intensity with phospho-specific antibodies was stronger after BHP fixation for every analyte tested. (B) Effect of long-term BHP fixation on protein antigenicity. Human colon was fixed for three months in BHP, followed by paraffin embedding. Specific and strong staining was observed for phospho-ERK Thr202/Tyr204 and cytokeratin 20, indicating retention of antigenicity for immunohistochemical studies (For description of histological details see Text S1).

In general, IHC and special chemistry staining after BHP fixation was equal to or stronger than formalin fixation, while specificity of staining remained equally high (Figures 10 and 11A, Figure S2, Tables S5 and S6, Text S1). This was especially apparent for the proliferation marker Ki-67, where proliferating human colon mucosal epithelial cells at the base of the crypt were intensely stained after fixation with BHP, compared to very poor staining after standard fixation with formalin. Cytokeratin 20 stained differentiated colon mucosal epithelial cells at the top of the crypt [30] and smooth muscle actin (SMA) localized in the smooth muscle layer of the colon mucosa, showed slightly stronger immunoreactivity in BHP fixed tissue. Similarly, there were a higher number of positively stained cells observed in uterine glands for estrogen receptor alpha and progesterone receptor after BHP fixation compared to formalin fixation. Periodic Acid Schiff stain showed excellent preservation of acidic mucin (blue) and polysaccharides (magenta) within the vacuoles of the colonic mucosal goblet cells [31]. To evaluate membrane protein staining we applied the HercepTest (Dako) to human ductal carcinoma in-situ (DCIS) and normal breast tissue. Tissue sections were less than 5 mm, and were immersed in a sufficient volume of fixative to allow adequate tissue penetration [20], [32]. Stronger membrane staining was observed in BHP than FFPE-fixed DCIS tissue, while normal tissue showed negative staining for both fixation chemistries (Figure 10, Text S1).

Our observation of more intense immunostaining in BHP compared to formalin fixed tissues raises the question of antigen-antibody specificity. This is especially true for clinical tests, such as the HercepTest for human Her2 abundance in breast cancer, where the clinical diagnosis has a profound impact on patient treatment and prognosis. To evaluate the specificity of the intense immunostaining seen in BHP-fixed tissue we performed a protein competition assay with the HercepTest antibody, in which the HercepTest antibody was incubated with full-length Her2 protein prior to analysis. No staining was visible after the protein competition, indicating that the more robust Her2 staining seen in BHP-fixed DCIS breast tissue is specific and not due to secondary antibody reactivity or non-specific interactions (Figure S2). Of all antigens investigated, only CDX2 showed a slightly reduced staining intensity based on independent pathologist observations (Tables S2 and S3).

We further evaluated the preservation of antigenicity for 6 labile phosphorylated proteins that are of special relevance to cancer biology: ERK Thr202/Tyr204, GSK3 α/β Ser21/Ser9, p38 MAPK Thr180/Tyr182, eIF4G Ser1108, Acetyl-CoA Carboxylase Ser79 and Bcl-2 Ser70 (Figure 11A (data for Bcl-2 not shown), Table S6, Text S1).

In each case staining was significantly stronger after BHP fixation compared to formalin fixation. In the cases of phospho-ERK Thr202/Tyr204 in human colon cancer, phospho-GSK3 α/β Ser21/Ser9 in human DCIS, phospho-p38 MAPK Thr180/Tyr182 in mouse brain cortex and phospho-acetyl-CoA carboxylase Ser79 in mouse brain hippocampus, staining was virtually absent in formalin fixed samples, but clearly visible in matched BHP fixed tissue.

To evaluate the effect of long term fixation with BHP on the retention of tissue antigenicity, we fixed human colon mucosa for three months in BHP prior to paraffin embedding. As demonstrated by strong staining for phospho-ERK Thr202/Tyr204 and cytokeratin 20, antigenicity for phosphorylated and non-phosphorylated proteins was apparently fully retained (Figure 11B, Text S1).

## Discussion

### Room temperature preservation of phosphoproteins is a necessity for molecular profiling

At the functional level cancer pathogenesis is influenced by differential activation/deactivation of cellular signaling networks that are enzymatically modulated by posttranslational modification of proteins by kinases and phosphatases. A high proportion of the current phase I–III drug pipeline for cancer therapy are protein kinase inhibitors [1]. It is therefore critical to measure the activity of kinases and phosphatases directly in human biopsy tissue in order to match the correct targeted thearpy with each patient's unique signaling architecture. Nevertheless, phosphoprotein biomarkers will not achieve their expected clinical impact unless the significant physiologic and technological barriers to proper phosphoprotein extraction, yield, sensitivity of detection, and stabilization are overcome.

Excised tissue is alive, and the cells therein experience a host of traumatic changes to their new environment, including the absence of vascular perfusion, ischemia, hypoxia, acidosis and accumulation of cellular waste. We have previously demonstrated that within 30 minutes after procurement protein phosphorylation changes dramatically in clinical tissue specimens [5]. Only the specific and rapid preservation of phosphoproteins can therefore yield information representative of the *in vivo* state of the signaling network within the tissue at the time of excision. Ideally this preservation should be compatible with morphologic examination and paraffin embedding similar to the standard practice of using FFPE. Such a preservation system will enable the same high degree of molecular and morphologic analysis to be applied to diagnostic samples as well as research samples. Rapid, controlled temperature elevation to inactivate enzymatic activity in tissue has been found to preserve phosphoproteins in mouse heart and brain analyzed by liquid-based techniques such as electrophoresis and mass spectrometry [33]–[35]. Although thermal treatment effectively inhibits enzymatic activity, it fails to preserve cellular morphology. Formalin, on the other hand, preserves tissue histomorphology accurately at room temperature in a paraffin block - but lacks adequate phosphoprotein preservation properties [14], [15]. These deficiencies of formalin are more than tissue-based penetration related issues as we have shown that formalin failed to preserve an EGF induced increase in the phosphorylation of EGFR Tyr1068 in suspension cells that are likely immediately fixed, thus indicating inadequate retention of phosphorylation due to the slow inhibition of phosphatase enzymes (Figure 5). One may assume that adding a phosphatase inhibitor to formalin could overcome this shortcoming. Unless phosphatases and kinases are adequately inhibited, retention of kinase activity, in the absence of phosphatase activity, will result in falsely elevated phosphoprotein levels [5].

Although alternative fixation chemistries to formalin have been developed [36]–[38], none address the specific need for one-step, room temperature preservation of phosphoproteins and tissue histomorphology in a *single* paraffin block. We designed a novel fixation chemistry, based on our previous observations related to the need to inhibit both phosphatases and kinases in tissue samples prior to tissue processing [5], [9]. The study presented herein demonstrates the feasibility of combining room-temperature preservation of phosphoproteins with retention of tissue histomorphology in a paraffin block (Table 1). This new fixative formulation also incorporated reagents to address less appreciated aspects of tissue fixation, such as infectious agent inactivation or attenuation, which can be accomplished with denaturing and cross-linking fixatives [37].

### Merits of current fixation/preservation methods

One advantage of snap-freezing over formalin fixation is the ease of protein extractability from frozen samples. Formalin fixation causes permanent cross-linkage between amino groups [39], which renders the extraction of full-length proteins very difficult. Using a standard protein extraction protocol for small tissue samples (e.g. laser capture microdissected material or core needle biopsy tissue sections) [19], mouse liver tissue fixed in formalin for one day followed by paraffin embedding yielded 13 times less protein per tissue area than matched snap-frozen tissue. In contrast, the total protein yield from tissue fixed with BHP for seven days followed by paraffin embedding was equal to snap-frozen tissue. Only after adopting a strong extraction buffer in a much longer extraction protocol were we able to increase the protein yield from FFPE tissue to levels with frozen tissue.

Another advantage of snap-freezing is the speed at which ongoing enzymatically-driven biochemical reactions are halted. Cellular kinase and phosphatase activity are rapidly inhibited (less than one minute) when cells are frozen in liquid nitrogen and the *in vivo* state of the cellular signaling architecture is not altered because of the change in the cellular environment. We speculated that the slow speed of formalin fixation could contribute to its limited success in preserving protein phosphorylation levels. Although formalin penetrates tissue at about 1 mm/h [40] the actual fixation process is much slower, requiring fixation times of eight hours or more, depending on tissue type and size [41]. We were able to show that protein tyrosine phosphatases are active for at least 15 minutes after inserting a small piece of breast tissue (1 mm^3^) into formalin. During this time the cell is reacting to *ex vivo* stresses, altering cell signaling patterns in reaction to the toxic stress of fixation. Supporting a direct effect of formalin fixation on phosphoprotein signaling, we found that an EGF-induced phosphorylation peak of EGFR Tyr1068 in U266 cells suspended in formalin, was not adequately preserved (Figure 5). This is in agreement with previous studies that have already indicated that the level of phosphoproteins is not adequately preserved in FFPE tissue [14], [15] and supports the concern that molecular analysis performed with FFPE tissue may skew phosphoprotein results.

In contrast to formalin, BHP fixation reduced the total tyrosine phosphatase activity in breast tissue to 18% after one minute of fixation, with no residual phosphatase activity observed by 15 minutes. This speed of phosphatase inhibition translated directly into the preservation of the expected increase in EGFR Tyr1068 phosphorylation after EGF stimulation of U266 cells. Even though the measurement sensitivity of reverse phase protein microarrays is very high (femtomolar range) [17], [22] no significant difference between snap-frozen tissue and BHP fixed, paraffin-embedded tissue was found in 9/10 phosphoproteins evaluated in paraffin embedded mouse liver, except for a slight increase in phospho-AMPKβ1 Ser108 (Figure 4C). AMPK is activated by cellular stress, such as hypoxia or ischemia, through an increased ratio of AMP:ATP (for review, see [42]). AMPK consists of a heterotrimer with a catalytic α unit and regulatory β and γ units. Phosphorylation at Ser108 of the β1 unit of AMPK is required for enzyme activation [43]. Its slight elevation could indicate a cellular response to the cytotoxic fixation process, or an improved exposure of the AMPK antigenic epitope. If AMPK elevation were due to a toxic reaction of the cell to BHP, we would expect that reactive changes would have been observed in other phosphoproteins. This was not the case.

We further demonstrated that the fixation process itself does not progressively alter phosphoprotein levels, even if samples were fixed for as long as one week before paraffin embedding. This is important, since the time from tissue collection to paraffin embedding may fluctuate over several days due to weekends, holidays, or the necessity to ship fixed samples to external facilities for processing. Also, inter-patient differences in tissue architecture did not significantly impact phosphoprotein preservation, as shown for two cases of highly labile human colon mucosa from two different patients that had nearly identical phosphoprotein patterns after fixation and paraffin embedding (Figure 4A).

Together, this supports our hypothesis that room temperature preservation of phosphoproteins and their intrinsic activation portraits in a paraffin block is possible. It is estimated that about 100–120 million proteins per cell are involved in signal transduction [44], [45], while about 33% of proteins are phosphorylated at any given time [45]. Here, we have focused on a subset of phosphoproteins selected to cover (i) several subcellular locations (membrane receptors, cytoplasmic proteins, cytoskeletal proteins and nuclear transcription factors), (ii) key functional signaling pathways (hypoxia, oxidative stress, growth/proliferation, apoptosis) and (iii) the amino acids at which the majority of phosphorylations occur (serine, threonine, tyrosine). We further based our selection on targets that were found to be reactive in previous phosphoprotein stability timecourse experiments [5]. Based on our current observations using the described representative selection of phosphoproteins, it is feasible to collect tissue using BHP for cell signaling based pathway mapping analysis – even when freezing is not an option. Future studies will be necessary to extend the evaluation of the described technology to additional phosphoprotein targets and signaling pathways.

### Clinical application requires preservation of tissue morphology and antigenicity

For clinical utility, a room-temperature phosphoprotein preservative has to overcome the limitations posed both by formalin (i.e. limited preservation of phosphoproteins and limited extractable biomolecule yield), and freezing (i.e. lack of availability in clinical laboratories and impairment of tissue morphology), while maintaining diagnostic quality morphology. We have compared BHP preservation of tissue morphology and antigenicity with FFPE, the most widely used preservative for tissue structure and cellular components [41]. In over 20 different human, mouse and feline tissues, BHP fixation showed equivalent preservation of morphology with full cytoplasmic and membrane detail, as well as better preservation of nuclear structures (chromatin and nucleoli) than matched FFPE tissues. Nuclear size was maintained in human colon mucosa epithelial cells, mouse brain Purkinje cells, mouse liver hepatocytes, and mouse pancreas islet of Langerhans cells. The finding that nuclear structure and size was equivalent to formalin fixed paraffin embedded tissue is a significant finding if the fixative is to be used for diagnostic pathology. Osmotic changes induced by alcohol-based fixatives can often cause cell shrinkage, thus limiting their application for diagnostic specimens. Modifications to alcohol based fixatives, such as addition of trehalose [46] or glycine [36] have mitigated some of these ethanol artifacts.

Professional socities such as the American Society of Clinical Oncologists (ASCO) and the College of American Pathologists (CAP) advocate standardized, reproducible immunohistochemical analysis of FFPE tissue samples for Her2, ER and PR clinical diagnositic assays [20], [32]. Although the Her2 testing guidelines stipulate using only FFPE tissue samples fixed for 6–48 hours, scant literature evidence exists for the exclusive use of FFPE samples or longer fixation times [47]–[49]. In our immunohistochemical and microarray comparisons between our biomarker and histology preservative and formalin, we did not adjust antibody concentrations (primary or secondary antibodies) or antigen retrieval methods. Some antibodies routinely used in IHC recognize cross-linked forms of the protein with higher affinity than native forms. Any future/current non-formalin fixation chemistry may benefit from optimization of reagent parameters.

Decalcification of bony tissues was a serendipitious discovery related to propeties of the biomarker and histology preservative. Decalcification is usually accomplished using strong acid (formic acid) or chelating agents (EDTA), which disrupts morphology, alters antigenicity, and is time consuming. Molecular profiling in bony tissues, such as bone metastasis or inner ear structures, has been hindered by the ability to measure phosphoproteins and nucleic acids in decalcified samples [25]–[28].

A primary goal of this study included making observations regarding preservation of key diagnostic immunohistochemical antigens. Ki-67, estrogen receptor alpha, progesterone receptor and HER2 where fully preserved in BHP fixed tissue. In fact, Ki-67 staining was greatly enhanced after BHP fixation. Ki-67 strongly stained proliferating human colon mucosal epithelial cells at the base of the crypt, whereas poor staining was observed after standard fixation in formalin processed in parallel with the BHP fixed tissue. Antigen-antibody specificity is an essential element of immunohistochemistry, therefore any fixative or fixation process must maintain the antigens in a state compatible with diagnostic assays. As shown using a Her2 antibody competition assay, HercepTest staining in BHP-fixed DCIS breast tissue was highly specific (Figure S2) demonstrating the utility for research specimens.

Of 14 non-phosphorylated protein antigens tested, only CDX2 showed a weaker than expected staining. All phosphoprotein antigens evaluated stained stronger after BHP fixation, which can greatly facilitate future phosphoprotein-targeted immunohistochemistry. For example, Acetyl-CoA Carboxylase Ser79 staining was visible in mouse brain hippocampus cell processes where no staining was seen after formalin fixation. In this instance, assessment of Acetyl-CoA Carboxylase phosphorylation levels would have been inaccurately described as negative if FFPE tissue had been analyzed.

### Conclusions

Preserving labile tissue phosphoproteins and full diagnostic histomorphology at room temperature in a paraffin block removes a critical roadblock to the realization of individualized therapy. Herein, we present data demonstrating that we have developed and successfully applied a formalin-free phosphoprotein preservation chemistry to room-temperature usage, combined with paraffin embedding. The special attribute of this technology is the combination of (i) the rapid stabilization of the phosphoprotein signature by simultaneous inhibition of kinase and phosphatase activity; (ii) the compatibility with standard paraffin embedding, obviating the need for freezing; (iii) the preservation of diagnostic histomorphology; (iv) the significantly improved yield of total and phosphoprotein analyte molecules extracted from the tissue; and (v) the compatibility with downstream analytical methods such as immunohistochemistry, reverse phase protein microarrays, Western blots and ELISA. This technology has the potential to facilitate individualized therapy based on the measurement of phosphorylation-driven cellular signaling events. Large-scale validation of this preservation technology is warranted to obtain cross platform comparisons with current methodologies and to establish its applicability to a wide range of phosphoprotein and immunohistochemical targets.

## Materials and Methods

### Ethics Statement

Animal studies were carried out in strict accordance with the recommendations in the Guide for the Care and Use of Laboratory Animals of the National Institutes of Health. The protocol was approved by the George Mason University institutional animal care and use committee (Number: 0176). All tissue samples were taken as a terminal event post euthanasia, and all efforts were made to minimize suffering. Feline testis tissue from a privately owned pet was obtained with owner's consent during a routine neutering operation.

Human surgical tissue specimens were collected from patients under written informed consent following the protocols by the Mediterranean Institute of Oncology Internal Ethical Review Board (samples 13–16 (Table S7)) or the institutional review boards of Inova Fairfax Hospital and George Mason University (all other samples (Table S7)). Tissue was excised in the surgical suite following standard of care guidelines. Tissue was transported at room temperature to the surgical frozen section room. A board-certified pathologist performed gross examination of each tissue sample and provided non-diseased and/or diseased tissue that was not required for diagnosis.

### Fixative preparation and use

The biomarker and histology preservative (BHP) was prepared from a combination of a precipitating fixative, a permeation enhancer, a carboxylic acid, phosphatase and kinase inhibitors, and reversible cross linkers (Table 1) (patent application PCT/US11/22463). In short, the carboxylic acid (Sigma-Aldrich) was solubilized in a water based buffer solution (Thermo Fisher Scientific). After addition of ethanol (Sigma-Aldrich), the permeation enhancers (Thermo Fisher Scientific), as well as phosphatase inhibitors (Calbiochem; Sigma-Aldrich) and kinase inhibitors (Sigma-Aldrich; Enzo Life Sciences) were mixed into the solution. Finally, reversible cross linkers (Thermo Fisher Scientific) were added to complete the fixative. The described fixative solution will be commercially available through Theranostics Health, Inc. To ensure reproducibility of the described data, the authors are happy to provide BHP for evaluation purposes prior to commercialization of the product (please contact Virginia Espina at vespina@gmu.edu or Claudius Mueller at cmuelle1@gmu.edu).

Tissue samples were immersed into the fixative and kept at room temperature until paraffin embedding (if applicable) (Table S7). Tissue preserved in BHP was processed using standard ethanol-based tissue processing protocols by commercial histology laboratories (AML Laboratories, Baltimore, MD or HSRL Mt. Jackson, VA) or a clinical diagnostic pathology laboratory (Mediterranean Institute of Oncology, Italy; samples 13–16 (Table S7)) with no contact to formalin-containing solutions. Tissue was processed directly from the BHP fixative without being placed in 70% ethanol as was the case with formalin fixed tissue samples. Samples were processed through graded ethanol (70%, 95%, and 100%), cleared in xylene, and embedded in Paraplast paraffin (Mercedes Medical) or McCormick Paraplast plus (Leica Microsystem; samples 13–16 (Table S7)). Tissue fixed in 10% neutral buffered formalin was processed through formalin and also embedded in Paraplast paraffin.

### Mouse tissue acquisition and preservation

Mouse tissue was collected from NOD SCID, C57BL/6, DBA/2J and ICR/CDI mouse strains. Mice were housed according to standard animal care procedures with water and chow provided *ad libitum*. After euthanasia with CO_2_, organs were dissected and tissues immersed in BHP, 10% neutral buffered formalin or snap-frozen in liquid nitrogen within 15 minutes of euthanasia (Table S7). Paraffin embedding and sectioning was performed at either AML Laboratories (Baltimore, MD, USA) or HSRL (Mt. Jackson, VA, USA). Tissue fixed in 10% neutral buffered formalin was processed through formalin, while BHP-fixed tissue was processed using standard ethanol-based tissue processing protocols with no contact to formalin-containing solutions.

### Human tissue acquisition and preservation

Patients undergoing surgical procedures in a community hospital (Inova Fairfax Hospital, Fairfax, VA or Mediterranean Institute of Oncology, Catania, Italy) were enrolled and written informed consent was obtained for participation in an IRB approved research protocol according to the Declaration of Helsinki. Tissue was excised in the surgical suite following standard of care guidelines. Tissue was transported at room temperature to the surgical pathology grossing room, where a board certified pathologist or physician assistant performed gross examination of each tissue sample and provided tissue that was not required for diagnosis. Tissue was cut into uniform pieces less than 5 mm×5 mm, and immediately submerged in BHP or 10% neutral buffered formalin. Tissue processing/embedding was performed on site using a Leica ASP 3000 automated system (samples 13–16 (Table S7)) or by a commercial histology service (AML Laboratories, Baltimore, MD or HSRL, Mt. Jackson, VA). Tissue fixed in 10% neutral buffered formalin was processed through formalin, while BHP-fixed tissue was processed using standard ethanol-based tissue processing protocols with no contact to formalin-containing solutions.

Human bone marrow core biopsies were collected following an IRB approved research protocol, with written informed consent, from patients undergoing a bone marrow aspirate as part of a diagnostic work-up for multiple myeloma. The core biopsy was collected from the posterior iliac crest using a trephine needle. The biopsy was placed immediately into an aliquot of BHP at room temperature.

### Protein extraction from frozen and paraffin embedded tissue

Whole slide lysates of paraffin embedded tissue were deparaffinized in two changes of xylene for 15 minutes each, rehydrated in graded alcohols (100%, 95%, 70%) and air dried prior to protein extraction. The tissue area was estimated by outlining a representative image of a serial section of each sample using a polygon drawing software option with the ArcturusXT laser capture microdissection instrument (Life Technologies, Carlsbad, CA) and calculating the resulting polygon area. Whole tissue sections were scraped off with a clean razor blade and added to a volume of extraction buffer based on the area of each tissue section. Frozen tissue was sectioned in a cryostat (5 µm) and directly added to a microcentrifuge tube with a volume of extraction buffer based on the area of the tissue section.

Two different extraction protocols were applied. Gentle extraction conditions (extraction protocol 1) consisted of an extraction buffer made of a 10% (v/v) solution of Tris(2-carboxyethyl)phosphine (TCEP; Pierce, Rockford, IL) in Tissue Protein Extraction Reagent (T-PER™, Pierce)/2X SDS Tris-glycine buffer (Invitrogen, Carlsbad, CA) [19]. Protein lysates were incubated at 100°C for 8 minutes. Strong extraction conditions (extraction protocol 2) were adapted from Ostasiewitz et al. [13]. In short, samples were incubated at 100°C for 60 minutes with vortexing every 10 minutes in an extraction buffer consisting of 4% SDS (Research Products International, Mt. Prospect, IL) and 100 mM DTT (Thermo Fisher Scientific, Pittsburgh, PA) in a 100 mM Tris-HCl solution (Bio-Rad, Hercules, CA) at pH 8. Samples were frozen at −80°C until analysis.

### Cell culture and cell lysis extracts

U266 cells were cultured to 80% confluence with appropriate medium supplemented with fetal bovine serum and spun at 300 x g for 5 minutes at room temperature. The cells were resuspended in 12 mL of medium and distributed equally into 3 aliquots. To measure unstimulated levels of phospho-epidermal growth factor receptor (EGFR) Tyr1068, 200 µL of cell suspension was spun at 800 x g for 3 minutes and washed with phosphate buffered saline without calcium or magnesium (PBS) (Gibco). This was repeated once and then either 100 µL of BHP or 10% neutral buffered formalin was added. For direct cell lysis, extraction protocol 1 was applied (see above). After fixation for 10 minutes or 2 hours extraction protocol 1 was applied to lyse cells. To stimulate epidermal growth factor (EGF) receptor phosphorylation cells were incubated with 200 ng/mL human EGF (Cell Signaling, Danvers, MA) for up to 30 minutes. Cell aliquots were removed from the suspension and either directly lysed or fixed and lysed as described above after 10 minutes and 30 minutes of stimulation. Protein extracts were frozen at −80°C until printing on reverse phase protein microarrays [17], [22].

### Reverse Phase Protein Microarray (RPMA) construction and staining

Reverse phase protein microarrays (RPMA) are a technology platform designed for quantitative, multiplexed analysis of cellular proteins. With very minimal volume requirements RMPA achieve high sensitivity (femtogram to attogram range) and good precision (<15% CV intra- as well as inter-assay), which makes them critical methodological components for several clinical trials [2]. Cellular/tissue lysates were printed on glass backed nitrocellulose array slides (Schott, Lexington, KY) using an Aushon 2470 arrayer (Aushon BioSystems, Burlington, MA) equipped with 350 µm or 185 µm pins as previously described [2]. Serial two-fold dilutions of bovine serum albumin (concentration 1.0 mg/mL) were printed on the arrays as a total protein standard. RPMA staining was performed on a Dako Autostainer per manufacturer's instructions (CSA kit, Dako). Each slide was incubated with a single primary antibody at room temperature for 30 minutes (for antibody details see Table S5). The ssDNA antibody microarrays and respective negative control (secondary antibody only) microarrays were baked for 2 hours at 80°C prior to blocking, with no Re-Blot (Millipore, Billerica, MA) incubation. Antibodies were validated by western blotting as previously described [18]. In addition a phospho-ERK Thr202/Tyr204 specific western blot was performed using frozen and BHP-fixed human colon mucosa to determine the compatibility of BHP fixation with downstream western blotting as well as unaltered phosphoprotein band morphology and specificity compared to frozen tissue (Figure S3). The negative control slide was incubated with antibody diluent. Secondary antibody was goat anti-rabbit IgG H+L (1∶10,000) (Vector Labs, Burlingame, CA). Subsequent signal detection was amplified via horseradish peroxidase mediated biotinyl tyramide deposition with chromogenic detection (Diaminobenzidine) per manufacturer's instructions (Dako). RPMA images were captured with a flat bed scanner at 600 dpi (350 µm pins) or 1200 dpi (185 µm pins) resolution. Signal intensity for each spot/array was determined with ImageQuant ver5.2 (GE Healthcare, Piscataway, NJ). RPMA intensity data was either normalized to total protein content or beta actin levels. Total protein per microarray spot was determined with Sypro Ruby protein blot stain (Invitrogen/Molecular Probes) per manufacturer's directions and imaged with a CCD camera (Nova Ray, Alpha Innotech, San Leandro, CA).

### Tyrosine phosphatase activity

Human breast tissue organoids, containing breast epithelium, adipose tissue and stromal elements, were cultured in serum-free DMEM/F12 as previously described (Espina et al PloS One April 2010). Organoids of about 1-2 mm^3^ size were removed from the culture flask and fixed for 1, 5 or 15 minutes in 500 µL of either 10% neutral buffered formalin or BHP. After fixation each organoid was washed 2 times in 1 mL of PBS (Gibco) to remove residual fixative. 250–500 µL of non-denaturing extraction buffer (20 mM Tris-HCl (pH 7.5) (Bio-Rad, Hercules, CA), 150 mM NaCl (Thermo Fisher Scientific, Pittsburgh, PA), 1 mM EDTA (Fluka, St. Louis, MO), 1 mM EGTA (Sigma-Aldrich, St. Louis, MO)) was added to each organoid depending on organoid size. Tissue was manually homogenized using a glass tissue grinder for 1 minute, followed by 1 minute of sonication. This process was repeated 3 times and the homogenate spun at 14,000 x g for 10 minutes at 4°C. The supernatant was divided into aliquots and frozen at −80°C until measurement.

Tyrosine phosphatase activity was measured using the RediPlate 96 EnzChek Tyrosine Phosphatase Assay Kit (Invitrogen, Carlsbad, CA) according to the manufacturer's protocol. Samples were measured in duplicate per assay and two independently prepared samples/organoids were analyzed for each time point and fixation type.

### Evaluation of Protein Extractability

Following euthanasia with CO_2_, brain and liver from 10 week old ICR/CDI mice (Harlan) were removed and sectioned into 4 pieces that were either snap-frozen in liquid nitrogen, fixed in BHP for 1 day, fixed in BHP for 7 days, fixed in 10% neutral buffered formalin (NBF) for 1 day or fixed in NBF for 7 days. Fixed tissue was paraffin embedded and sectioned (5 µm thickness). Snap frozen tissue was kept at −80°C until sectioning in a cryostat (5 µm thickness). The tissue area was estimated by outlining a representative image of a serial section of each sample using a polygon drawing software option with the ArcturusXT laser capture microdissection instrument (Life Technologies, Carlsbad, CA) and calculating the resulting polygon area. Whole tissue sections were scraped off with a clean razor blade and added to a volume of extraction buffer based on the area of each tissue section. Protein extraction was performed using extraction protocol 1 and 2 (see above) and total protein content measured using a Sypro Ruby Protein Blot stain of a reverse phase protein microarray.

### Immunohistochemistry

Formalin fixed (FFPE) or BHP-Tissue fixed, paraffin embedded tissue sections (5 µm thickness) mounted on positively charged glass slides were baked at 56°C for 30 min, deparaffinized in xylene and rehydrated in a series of graded alcohols (100%, 95%, and 70%) with a final rinse in wash buffer (Dako). Immunostaining post heat induced epitope retrieval (for details on antibodies and respective epitope retrieval methods see Table S6) was performed on a Ventana BenchMark slide stainer with an Ultraview Universal DAB detection IVD kit (Ventana Medical System; samples 13–16 (Table S7)), a Leica BOND-MAX with a Bond Polymer Refine Detection kit (Leica Microsystems; sample 6 (Table S7)), or a Dako Autostainer with an EnvisionSystem+HRP staining kit (Dako; remaining samples (Table S7)) with development in diaminobenzidine or per manufacturer's instructions. Tissue sections were nuclear counterstained with Mayer's hemalume (Kaltek Srl; samples 13–16 (Table S7)), Harris Haematoxylin Acidified Papanicolaou stain (Modification II, CellPath; sample 6 (Table S7)), or hematoxylin (Dako; remaining samples (Table S7)) and Scott's Tap Water Substitute, and cover slips were applied with aqueous mounting medium (Faramount, Dako).

Periodic Acid Schiff staining (Richard-Allan Scientific, Kalamazoo, MI) of FFPE and BHP fixed colon mucosa sections was performed per manufacturer's instructions. The HecepTest (Dako) was performed according to the manufacturer's instructions. Images were captured with an Olympus BX51 microscope using 20x or 100x objectives.

To address the question of specificity for the increased staining seen in BHP-fixed samples as compared to equivalent tissues fixed in formalin we performed a protein competition assay with the Dako HercepTest Her2 antibody. DCIS breast tissue of a 48 year old female was collected after surgery and fixed for seven days in BHP, followed by paraffin embedding. Directly following deparaffinization, tissue sections were stained using the HercepTest kit (Dako). Very robust staining of the BHP-fixed as well as formalin-fixed tissue made heat induced epitope retrieval unnecessary. The tissue was blocked for one hour with the HercepTest kit rabbit serum IgG following the peroxidase block step. To compete the antibody prior to staining ready-to-use Her2 antibody from the kit was pre-incubated with 0.012 ng/ìl full length recombinant human Her2 protein (Origene) on a shaker at room-temperature for three hours. Following incubation, the antibody-protein mixture was centrifuged for 15 minutes at 16,000 g at room-temperature and the supernatant used directly for immunostaining following manufacturer's directions. Non-competed Her2 antibody was treated alongside the competed Her2 antibody without addition of the Her2 protein and immunostaining with both antibodies was performed side-by-side on a Dako Autostainer (Dako) using the same staining protocol.

### Comparison of nuclear size

Human colon mucosa and mouse brain, liver and pancreas tissue was fixed in 10% neutral buffered formalin or BHP, paraffin embedded and sections were de-paraffinized in xylene, rehydrated in graded alcohol and stained with hematoxylin and eosin. Using the Arcturus^XT^(Life Technologies) polygon drawing software, the area of 50 to 100 nuclei was measured per tissue and fixation type.

### Statistical Analysis

Standard error of the mean (SEM) was calculated for individual samples. The Mann-Whitney test was used to determine statistical significance between fixation times (Figure 3), while Student's t-test was used to determine statistical significance between nuclear sizes (Figure 9). P values <0.05 were considered significant. Due to the number of biological replicates (n = 3) for the comparison of phosphoprotein levels between BHP fixed and snap-frozen tissue (Figure 4C) the significance of differences was estimated by the amount of SEM overlap. Non-overlapping SEM was used as an indicator of possible significant difference [50].

## Supporting Information

Figure S1 **Tissue size has no significant effect on phosphoprotein preservation in BHP-fixed, paraffin-embedded human colon mucosa.** Human colon mucosa was fixed for two days (A) or seven days (B) in BHP, followed by paraffin embedding. After sectioning, the tissue area was measured using the Arcturus^XT^ platform and protein extraction buffer added to maintain a constant buffer/tissue area ratio. Protein extracts were printed and the abundance of 11 phosphoproteins measured using reverse phase protein microarrays. Relative phosphoprotein abundance is plotted versus respective tissue area. No statistically significant correlation (p>0.05) was found to exist between any phosphoprotein and tissue area as measured by Spearman's Rho coefficient (Table S1).
(TIF)

Figure S2 **HercepTest specificity in BHP-fixed human breast DCIS as demonstrated by protein-antibody competition assay.** Herceptest specificity in BHP-fixed human breast DCIS as demonstrated by protein competition assay. DCIS breast tissue of a 48 year old female was collected after surgery and fixed for seven days in BHP, followed by paraffin embedding. Directly following deparaffinization, tissue sections were stained using the HercepTest kit (Dako). Very robust staining of the BHP-fixed as well as formalin-fixed tissue made heat induced epitope retrieval unnecessary. The tissue was blocked for one hour with the HercepTest kit rabbit serum IgG following the peroxidase block step. To compete the antibody prior to staining ready-to-use Her2 antibody from the kit was pre-incubated with 0.012 ng/µl full length recombinant human Her2 protein (Origene) on a shaker at room-temperature for three hours. Following incubation, the antibody-protein mixture was centrifuged for 15 minutes at 16,000 g at room-temperature and the supernatant used directly for immunostaining following manufacturer's directions. Non-competed Her2 antibody was treated alongside the competed Her2 antibody without addition of the Her2 protein and immunostaining with both antibodies was performed side-by-side on a Dako Autostainer (Dako) using the same staining protocol. No staining was visible after competing the Her2 antibody with full length Her2, indicating that the increased staining seen in BHP-fixed tissue compared to formalin fixation is specific.
(TIF)

Figure S3 **Phospho-ERK Thr202/Tyr204 western blot of frozen and BHP-fixed human colon mucosa.** Human colon mucosa was collected after surgery (colon a  =  male, colon b  =  female) and snap frozen or fixed in BHP prior to paraffin embedding. Following, samples were lysed and proteins separated in a 4–20% Tris-Glycine gel (Invitrogen). After blotting the membrane was stained using a phospho-ERK Thr202/Tyr204 antibody (Cell Signaling), stripped and re-probed with a beta actin antibody (Cell Signaling). Lysates from BHP-fixed tissue demonstrated the specific phospho-ERK double band (42/44 kDa) also seen in the frozen sample.
(TIF)

Table S1 **Spearman's rho analysis of tissue size versus phosphoprotein preservation in BHP-fixed, paraffin-embedded human colon mucosa.**
(DOC)

Table S2 **Immunohistochemical Evaluation of Human Colon Mucosa – Pathologist 1.**
(DOC)

Table S3 **Immunohistochemical Evaluation of Human Colon Mucosa – Pathologist 2.**
(DOC)

Table S4 **Human/Animal tissues collected and fixed in biomarker and histology preservative.**
(DOC)

Table S5 **Validated primary antibodies used for reverse phase protein microarrays.**
(DOC)

Table S6 **Antibodies used for immunohistochemistry.**
(DOC)

Table S7 **Tissue sample identification and fixation detail for each experimental procedure.**
(XLS)

Text S1 **Histology Description for H&E stained tissues and Immunohistochemistry.**
(DOC)
