# Supplementary figures and images for: One-Step Preservation of Phosphoproteins and Tissue Morphology at Room Temperature for Diagnostic and Research Specimens
Source: PLoS One. 2011 Aug 17;6(8):e23780. doi: 10.1371/journal.pone.0023780 (PMC3157466; doi:10.1371/journal.pone.0023780)

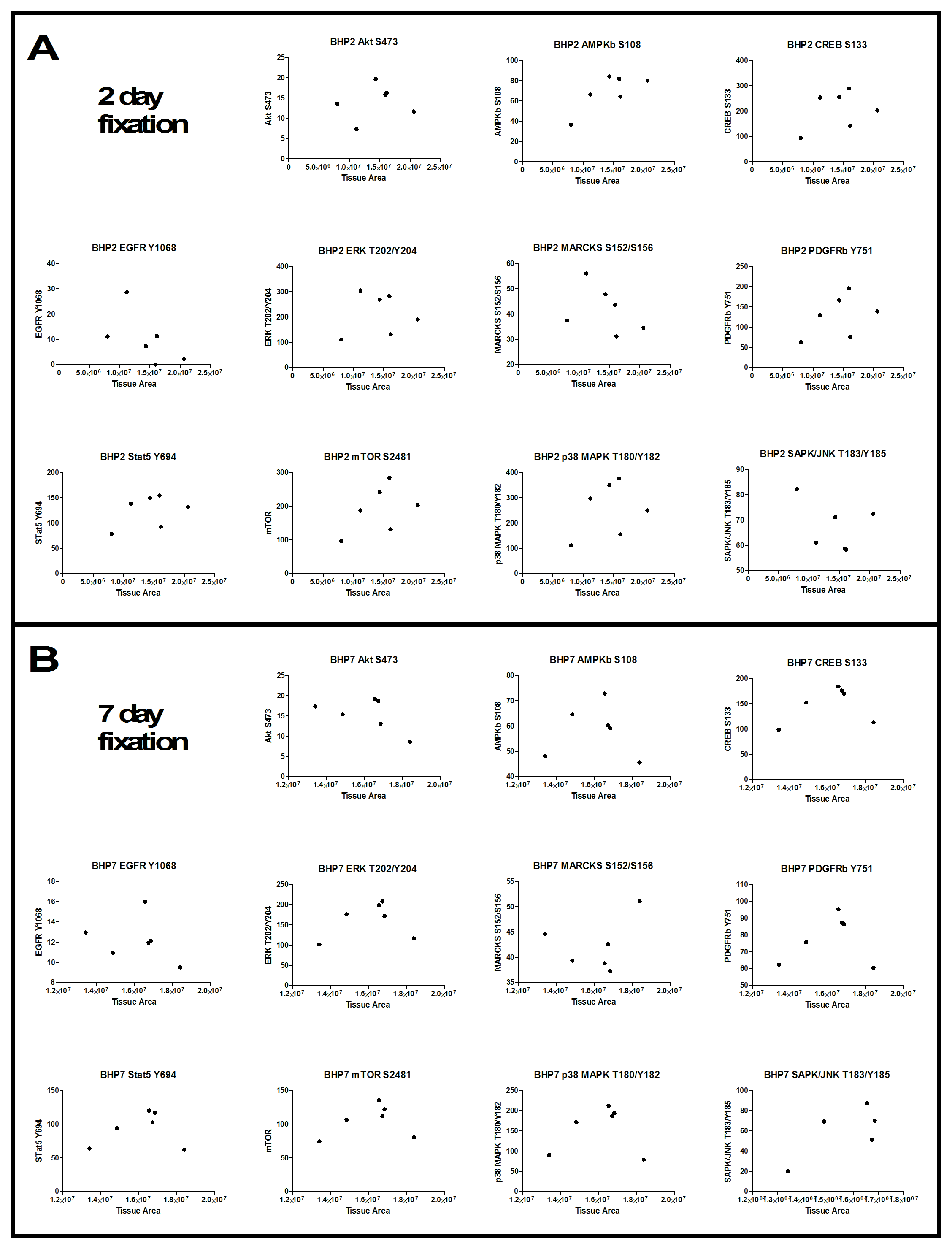

Supplement: Figure S1 — Tissue size has no significant effect on phosphoprotein preservation in BHP-fixed, paraffin-embedded human colon mucosa. Human colon mucosa was fixed for two days (A) or seven days (B) in BHP, followed by paraffin embedding. After sectioning, the tissue area was measured using the ArcturusXT platform and protein extraction buffer added to maintain a constant buffer/tissue area ratio. Protein extracts were printed and the abundance of 11 phosphoproteins measured using reverse phase protein microarrays. Relative phosphoprotein abundance is plotted versus respective tissue area. No statistically significant correlation (p>0.05) was found to exist between any phosphoprotein and tissue area as measured by Spearman's Rho coefficient (Table S1). (TIF) [file pone.0023780.s001.tif]

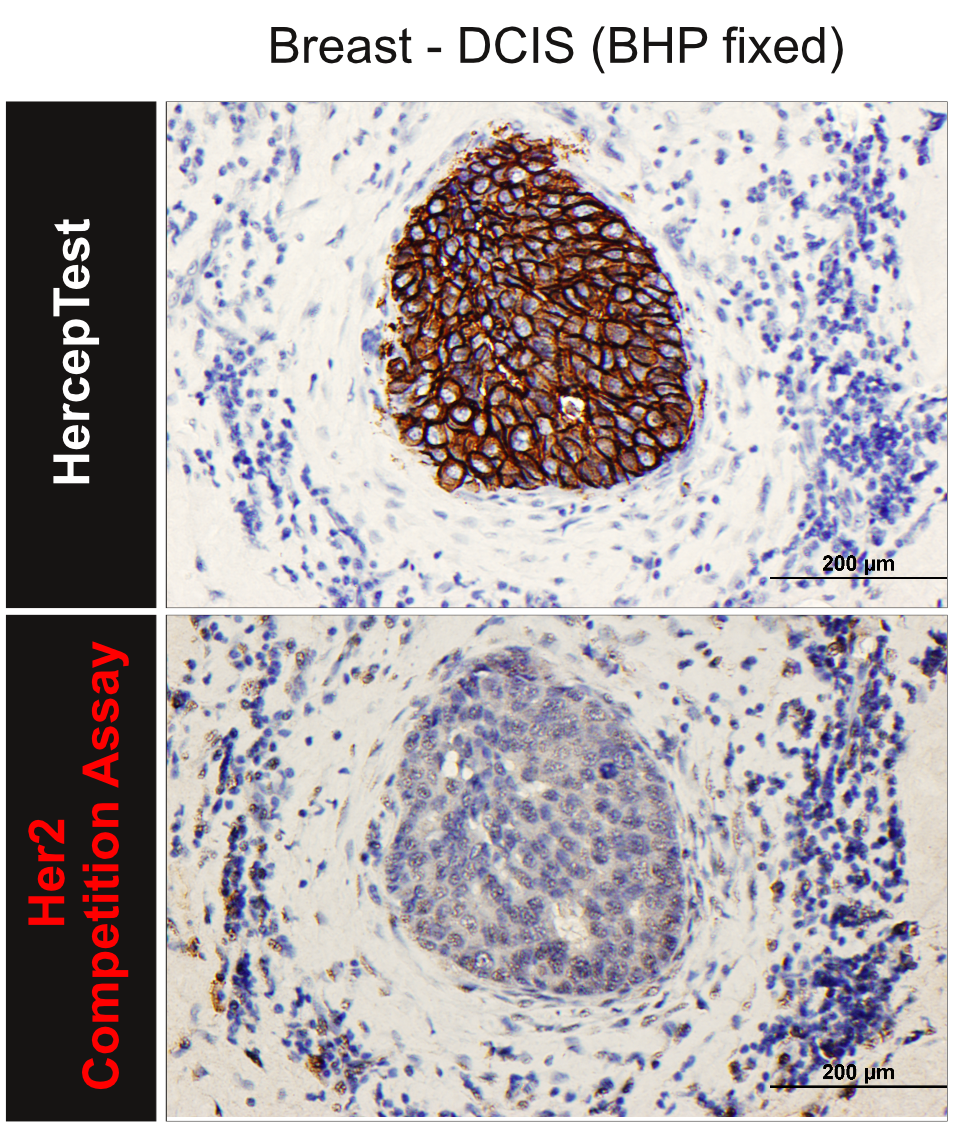

Supplement: Figure S2 — HercepTest specificity in BHP-fixed human breast DCIS as demonstrated by protein-antibody competition assay. Herceptest specificity in BHP-fixed human breast DCIS as demonstrated by protein competition assay. DCIS breast tissue of a 48 year old female was collected after surgery and fixed for seven days in BHP, followed by paraffin embedding. Directly following deparaffinization, tissue sections were stained using the HercepTest kit (Dako). Very robust staining of the BHP-fixed as well as formalin-fixed tissue made heat induced epitope retrieval unnecessary. The tissue was blocked for one hour with the HercepTest kit rabbit serum IgG following the peroxidase block step. To compete the antibody prior to staining ready-to-use Her2 antibody from the kit was pre-incubated with 0.012 ng/µl full length recombinant human Her2 protein (Origene) on a shaker at room-temperature for three hours. Following incubation, the antibody-protein mixture was centrifuged for 15 minutes at 16,000 g at room-temperature and the supernatant used directly for immunostaining following manufacturer's directions. Non-competed Her2 antibody was treated alongside the competed Her2 antibody without addition of the Her2 protein and immunostaining with both antibodies was performed side-by-side on a Dako Autostainer (Dako) using the same staining protocol. No staining was visible after competing the Her2 antibody with full length Her2, indicating that the increased staining seen in BHP-fixed tissue compared to formalin fixation is specific. (TIF) [file pone.0023780.s002.tif]

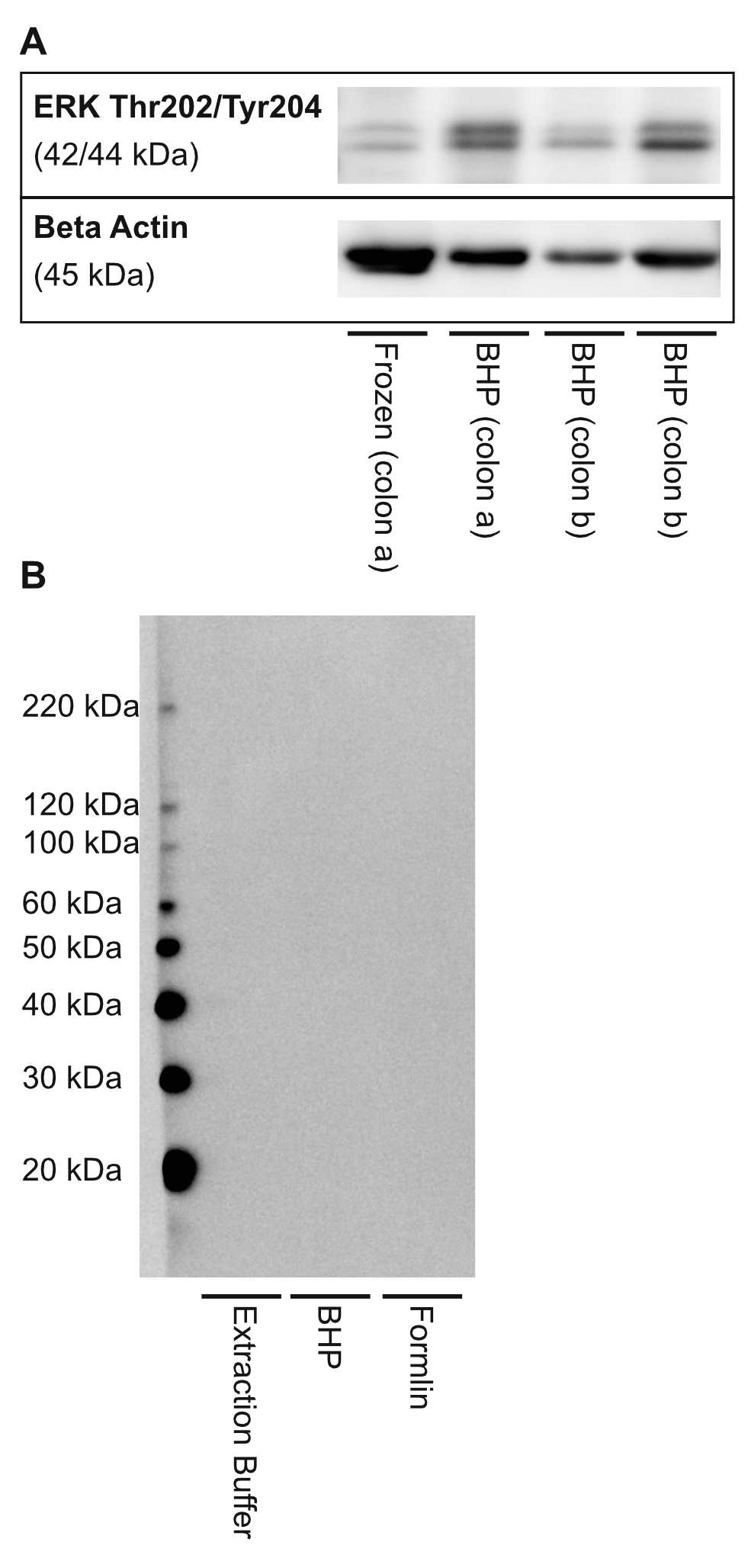

Supplement: Figure S3 — Phospho-ERK Thr202/Tyr204 western blot of frozen and BHP-fixed human colon mucosa. Human colon mucosa was collected after surgery (colon a = male, colon b = female) and snap frozen or fixed in BHP prior to paraffin embedding. Following, samples were lysed and proteins separated in a 4–20% Tris-Glycine gel (Invitrogen). After blotting the membrane was stained using a phospho-ERK Thr202/Tyr204 antibody (Cell Signaling), stripped and re-probed with a beta actin antibody (Cell Signaling). Lysates from BHP-fixed tissue demonstrated the specific phospho-ERK double band (42/44 kDa) also seen in the frozen sample. (TIF) [file pone.0023780.s003.tif]
