# Supplementary material for: One-Step Preservation of Phosphoproteins and Tissue Morphology at Room Temperature for Diagnostic and Research Specimens
Source: PLoS One. 2011 Aug 17;6(8):e23780. doi: 10.1371/journal.pone.0023780 (PMC3157466; doi:10.1371/journal.pone.0023780)
Supplement: Table S1 — Spearman's rho analysis of tissue size versus phosphoprotein preservation in BHP-fixed, paraffin-embedded human colon mucosa. (DOC) [file pone.0023780.s004.doc]

**Table S1.** Spearman's Rho analysis of tissue size versus phosphoprotein preservation in BHP-fixed, paraffin-embedded human colon mucosa.

|  | **2 day fixation** | | **7 day fixation** | |
| --- | --- | --- | --- | --- |
| **Phosphoprotein** | **Spearman's Rho Coefficient** | **p value** | **Spearman's Rho Coefficient** | **p value** |
| Akt S473 | 0.14 | 0.79 | -0.54 | 0.27 |
| AMPKbS108 | 0.31 | 0.54 | -0.37 | 0.47 |
| CREB S133 | 0.14 | 0.79 | 0.14 | 0.79 |
| EGFR Y1068 | -0.43 | 0.40 | 0.09 | 0.87 |
| ERK T202/Y204 | -0.03 | 0.96 | 0.03 | 0.96 |
| MARCKS S152/156 | -0.60 | 0.21 | -0.03 | 0.96 |
| mTOR S2481 | 0.37 | 0.47 | -0.09 | 0.87 |
| p38 MAPK T180/Y182 | 0.37 | 0.47 | 0.20 | 0.70 |
| PDGFRb Y751 | 0.37 | 0.47 | 0.26 | 0.62 |
| SAPK/JNK T183/Y185 | -0.37 | 0.47 | 0.26 | 0.62 |
| Stat5 Y694 | 0.14 | 0.79 | -0.03 | 0.96 |

For experimental details see Figure S1.
