# Supplementary material for: One-Step Preservation of Phosphoproteins and Tissue Morphology at Room Temperature for Diagnostic and Research Specimens
Source: PLoS One. 2011 Aug 17;6(8):e23780. doi: 10.1371/journal.pone.0023780 (PMC3157466; doi:10.1371/journal.pone.0023780)
Supplement: Table S2 — Immunohistochemical Evaluation of Human Colon Mucosa – Pathologist 1. (DOC) [file pone.0023780.s005.doc]

Table S2. Immunohistochemical Evaluation of Human Colon Mucosa – Pathologist 1.

|  | **BHP** | | | **Formalin** | | |
| --- | --- | --- | --- | --- | --- | --- |
| **Immunostain** | **Staining**  **Strength** | **Staining**  **Amount** | **Staining**  **Background** | **Staining**  **Strength** | **Staining**  **Amount** | **Staining**  **Background** |
| AE1AE3 | 2 | 2 | 2 | 3 | 2 | 3 |
| CK 20 | 3 | 2 | 2 | 3 | 3 | 3 |
| CDX2 | 1 | 3 | 2 | 3 | 3 | 3 |
| DPAS | 3 | 3 | 3 | 3 | 3 | 3 |
| CD3 | 3 | 3 | 3 | 3 | 3 | 2 |
| CD20 | 3 | 3 | 2 | 3 | 3 | 2 |
| CD34 | 3 | 3 | 3 | 3 | 3 | 3 |
| CD31 | 2 | 3 | 1 | 3 | 3 | 3 |
| CD38 | 2 | 3 | 2 | 2 | 3 | 1 |
