# Supplementary material for: One-Step Preservation of Phosphoproteins and Tissue Morphology at Room Temperature for Diagnostic and Research Specimens
Source: PLoS One. 2011 Aug 17;6(8):e23780. doi: 10.1371/journal.pone.0023780 (PMC3157466; doi:10.1371/journal.pone.0023780)
Supplement: Table S4 — Human/Animal tissues collected and fixed in biomarker and histology preservative. (DOC) [file pone.0023780.s007.doc]

**Table S4.** Human/Animal tissues collected and fixed in biomarker and histology preservative.

| **Mouse** | | | **Human** | **Feline** |
| --- | --- | --- | --- | --- |
| Brain | Liver | Small Intestine | Breast (Normal + DCIS) | Testis |
| Colon | Lung | Spleen | Bone Marrow |  |
| Ear (Cartilage) | Mammary | Stomach | Colon Mucosa (Normal + Cancer) |  |
| Eye | Ovary | Tail | Diffuse Large B-Cell Lymphoma |  |
| Fallopian Tube | Pancreas | Tongue | Lymph Node Met. from Cln. Cancer |  |
| Femur | Rib |  | Prostate |  |
| Heart | Salivary Glands |  | Reactive Lymph Node |  |
| Kidney | Sciatic Nerve |  | Uterus |  |
