# Supplementary material for: One-Step Preservation of Phosphoproteins and Tissue Morphology at Room Temperature for Diagnostic and Research Specimens
Source: PLoS One. 2011 Aug 17;6(8):e23780. doi: 10.1371/journal.pone.0023780 (PMC3157466; doi:10.1371/journal.pone.0023780)
Supplement: Table S5 — Validated primary antibodies used for reverse phase protein microarrays. (DOC) [file pone.0023780.s008.doc]

**Table S5.** Validated primary antibodies used for reverse phase protein microarrays.

| **Antibody** | **Vendor** | **Function** |
| --- | --- | --- |
| AKT | Cell Signaling Technology | Growth/Prosurvival |
| AKT Ser473 | Cell Signaling Technology | Growth/Prosurvival |
| AKT Thr308 | Cell Signaling Technology | Growth/Prosurvival |
| AMPK α1 Ser485 | Cell Signaling Technology | Hypoxia/Stress |
| AMPK β1 Ser108 | Cell Signaling Technology | Hypoxia/Stress |
| Beta Actin | Cell Signaling Technology | Cytoskeletal |
| Biliverdin Reductase Alpha | Abnova | Stress/Heme Degradation |
| Cleaved Caspase 3 Asp175 | Cell Signaling Technology | Apoptosis |
| Cleaved Caspase 9 Asp330 | Cell Signaling Technology | Apoptosis |
| CREB Ser133 | Cell Signaling Technology | Stress/Survival |
| E-Cadherin | Cell Signaling Technology | Motility/Adhesion |
| EGFR | Cell Signaling Technology | Growth Factor Receptor |
| EGFR Tyr1068 | Cell Signaling Technology | Growth Factor Receptor |
| eNOS Ser1177 | Cell Signaling Technology | Hypoxia/Stress |
| ERK | Cell Signaling Technology | Growth/Prosurvival |
| ERK Thr202/Tyr204 | Cell Signaling Technology | Growth/Prosurvival |
| Hemeoxygenase 1 | Stressgen | Stress/Heme Degradation |
| Her2 Tyr1248 | Cell Signaling Technology | Growth Factor Receptor |
| IRS1 Ser162 | Cell Signaling Technology | Glucose Metabolism |
| L13a | Cell Signaling Technology | Ribosom |
| MARCKS Ser152/Ser156 | Cell Signaling Technology | Motility/Adhesion |
| MRPL11 | Cell Signaling Technology | Mitochondrial Ribosom |
| mTOR Ser2481 | Cell Signaling Technology | Growth/Prosurvival |
| p38 MAPK Thr180/Tyr182 | Cell Signaling Technology | Stress/Inflammation |
| p53 | Cell Signaling Technology | Apoptosis |
| p53 Ser15 | Cell Signaling Technology | Apoptosis |
| PDGFR β | Cell Signaling Technology | Growth Factor Receptor |
| PDGFR β Tyr751 | Cell Signaling Technology | Growth Factor Receptor |
| PTEN | Cell Signaling Technology | Growth/Prosurvival |
| PTEN Ser380 | Cell Signaling Technology | Growth/Prosurvival |
| SAPK/JNK Thr183/Tyr185 | Cell Signaling Technology | Stress/Inflammation |
| ssDNA | Immuno-Biological Laboratory | DNA |
| Stat3 Ser727 | Cell Signaling Technology | Stress/Inflammation |
| Stat5 Tyr694 | Cell Signaling Technology | Stress/Inflammation |
| Sumo 1 | Cell Signaling Technology | Sumoylation |
| Tubulin | Cell Signaling Technology | Cytoskeletal |
