# Supplementary material for: One-Step Preservation of Phosphoproteins and Tissue Morphology at Room Temperature for Diagnostic and Research Specimens
Source: PLoS One. 2011 Aug 17;6(8):e23780. doi: 10.1371/journal.pone.0023780 (PMC3157466; doi:10.1371/journal.pone.0023780)
Supplement: Table S6 — Antibodies used for immunohistochemistry. (DOC) [file pone.0023780.s009.doc]

Table S6. Antibodies used for immunohistochemistry.

| **Antibody** | **Vendor** | **PK** | **pH** | **Phospho-Specific** |
| --- | --- | --- | --- | --- |
| Ki-67 (clone: MIB-1) | Dako | - | high | - |
| Cytokeratin 20 (clone: Ks20.8) | Dako | - | high | - |
| Smooth Muscle Actin (clone: 1A4) | Dako | - | high | - |
| Estrogen Receptor alpha (clone: 1D5) | Dako | - | 9 | - |
| Progesterone Receptor (clone: PgR 626) | Dako | - | 9 | - |
| Her2 (HercepTest) | Dako | - | - | - |
| AE1/AE3 | Dako | - | 6 | - |
| CD3 (clone: 2GV6) | Ventana | - | 8 | - |
| CD20 (clone: L26) | Ventana | - | 8 | - |
| p63 (clone: 4A4) | Ventana | - | 8 | - |
| PSA (clone: ER-PR) | Ventana | - | 8 | - |
| High MW Cytokeratin (clone: 34BE12) | Ventana | yes | 8 | - |
| CDX2 (clone: EPR2764Y) | Ventana | - | 8 | - |
| CD34 | Dako | - | 6 | - |
| ERK Thr202/Tyr204 | CellSignal | - | 9 | yes |
| GSK3 αβSer21/9 | CellSignal | - | 9 | yes |
| p38 MAPK Thr180/Tyr182 | CellSignal | - | 9 | yes |
| eIF4G Ser1108 | CellSignal | - | 6 | yes |
| Bcl-2 Ser70 | CellSignal | - | 6 | yes |
| AcetylCoA Carboxylase Ser79 | CellSignal | - | 9 | yes |

Formalin-fixed and BHP-fixed tissue sections were treated side-by-side using the same experimental conditions. PK = Proteinase K used; pH = pH of heat induced epitope retrieval method (20-30 minutes at 80°C)
