# Supplementary material for: One-Step Preservation of Phosphoproteins and Tissue Morphology at Room Temperature for Diagnostic and Research Specimens
Source: PLoS One. 2011 Aug 17;6(8):e23780. doi: 10.1371/journal.pone.0023780 (PMC3157466; doi:10.1371/journal.pone.0023780)
Supplement: Text S1 — Histology Description for H&E stained tissues and Immunohistochemistry. (DOC) [file pone.0023780.s011.doc]

# Text S1. Histology Description for H&E stained tissues and Immunohistochemistry

# Figure 6

**Feline Testes:** Seminiferous tubules. Clear delineation of nuclear chromatin and chromosomes in primary spermatocytes in BHP compared to FFPE.

# Mouse

**Purkinje Cells:** Cerebellar cortex ganglionic layer. Insert Purkinje cells with dendrites: Clear delineation of nuclear structure and cell body in BHP compared to FFPE

**Cortex-Neurons:** Cerebral Cortex: Insert Neuronal cells with dendrites: Nuclear structure and cell body delineated in BHP compared to FFPE.

Hippocampus: Pyramidal neurons Insert comparison of cell body and nuclear definition in BHP compared to FFPE

**Eye Retina:** Pigment epithelium, outer nuclear layer and inner nuclear layer. Insert compares nuclear delineation and nucleoli in BHP compared to FFPE.

**Ear Cartilage:** Elastic cartilage of the ear. Equivalent chondrocyte size and nuclear structure in BHP compared to FFPE.

**Tongue:** Tongue squamous epithelium, lamina propria and underlying skeletal muscle. Comparable histomorphology of BHP and FFPE.

**Sublingual Gland:** Gland parenchyma. Insert compares nuclear definition of epithelium for BHP compared to FFPE.

**Submandibular Gland:** Gland parenchyma. Insert compares epithelial nuclear structure and glandular definition for BHP compared to FFPE.

Liver parenchyma periportal region. Insert hepatocytes. Clear nuclear membrane delineation and prominent nucleoli in BHP compared to FFPE

**Heart myocardium:** Myocardial fibers with central nuclei and cross striations. Comparison of BHP and FFPE.

**Lung:**  Parenchyma alveoli and bronchioles. Equivalent morphologic definition for BHP and FFPE

# Figure 7 (Mouse Tissue)

**Kidney proximal tubules and glomeruli:** Insert: single glomerulus. Equivalent Bowman’s space and delineation of capillary loops for BHP versus FFPE.

**Pancreas acinar tissue and scattered islets of Langerhans:** Insert: single islet of Langerhans, alpha and beta endocrine cells. Comparable cellular and nuclear definition for BHP and FFPE.

**Spleen white pulp lymphocytes:** Nuclear membrane and nucleoli of lymphocytes are more prominent in BHP compared to FFPE.

**Stomach:** Gastric glands. Equivalent nuclear membrane, nucleoli and chromatin staining in BHP compared to FFPE.

**Small Intestine:** Mucosa and lamina propria: Insert compares nuclear chromatin structure for BHP compared to FFPE.

**Colon mucosa and lamina propria:**  Insert compares nuclear chromatin structure for BHP compared to FFPE.

**Mammary Gland:** Duct epithelial and myoepithelial cells with surrounding fibrous stroma and adipose cells. Dense nuclear chromatin staining in duct epithelium in BHP compared to FFPE

**Fallopian Tube columnar epithelium:** Insert demonstrates enhanced nuclear membrane delineation of columnar epithelium in BHP compared to FFPE.

**Ovary follicles with ooctyes and surrounding granulosa cells:** Comparable histomorphology of BHP compared to FFPE.

**Tail:** Cross section with central bone. Processed without separate decalcification. BHP fixation preserves bone morphology compared to fractured bone in FFPE.

**Rib bone, cartilage and bone marrow:** Full histomorphologic preservation with BHP without decalcification.

**Femur bone, cartilage and bone marrow:** Full histomorphologic preservation with BHP without decalcification. Insert megakaryocte in marrow hematopoetic cells.

Sciatic Nerve Schwann cells and nerve fibers. Insert showing preservation of Schwann cell nuclear structure.

# Figure 8 (Human Tissues)

**Breast duct epithelium and myoepithelium:** Insert: duct epithelium demonstrating enhanced nuclear chromatin in BHP compared to formalin.

**Uterus:** endometrium. Insert demonstrates inhanced nuclear chromatin staining in glandular epithelial nuclei in BHP compare to FFPE.

**Colon mucosa and lamina propria:**  Insert compares nuclear chromatin structure for BHP compared to FFPE. Nuclei are slightly larger in BHP compared to FFPE

**Bone marrow:** BHP without separate decalcification. Insert shows multinucleated megakaryocytes.

**Lymph node (reactive):** Follicular structure is comparable for BHP and FFPE.

Prostate: Glands and fibromuscular stroma. Glandular columnar epithelium is equivalent in BHP compared to FFPE, with enhanced nuclear chromatin staining in BHP.

# Figure 10 (Human Tissues)

**Colon Ki-67 staining:** Prominent staining of dividing cells at the crypt base for BHP compared to FFPE.

**Bone marrow:** Ki-67 nuclear staining of dividing hematopoetic precursors.

**Colon Cytokeratin 20:** Enhanced staining of differentiated outer zone of crypt for BHP compared to FFPE.

**Colon Smooth Muscle Actin:** Delineation of glandular stroma with equivalent staining intensity for BHP compared to FFPE.

**Uterus Estrogen Receptor (ER):** endometrial glands exhibit strong nuclear staining (insert) in BHP compared to FFPE.

**Uterus Progesterone Receptor (PR)**: endometrial glands exhibit strong nuclear staining (insert) in BHP compared to FFPE.

**Breast Ductal carcinoma in Situ (DCIS) Her2:** Strong homogeneous membrane staining of DCIS ductal epithelium with no staining of surrounding stroma. Enhanced intensity of BHP staining compared to FFPE.

**Breast Normal Ducts:** Her2 negative control.

**Colon Periodic Acid Schiff stain (PAS):** Equivalent staining of intralumenal polysaccharide for BHP compared to FFPE

**Colon AE1AE3:** Equivalent staining of mucosal glandular epithelium for BHP compared to FFPE.

# BHP fixed tissues only

**Diffuse Large B Cell Lymphoma:** Strong CD3 staining of neoplastic cells.

**Reactive Lymph Node:** Strong CD3 staining of lymphocytes.

**Lymph node metastasis CDX2:** Weakly positive staining of carcinoma cell cluster.

**Prostate:** Strong p63 staining of glands.

**Prostate:** Strong PSA staining of glands

**Prostate:** Clearly delineated glands by high MW cytokeratin staining.

**Diffuse Large Cell Lymphoma:** Strong CD20 staining of neoplastic cells

**Reactive Lymph Node:** Strong CD20 staining of follicles.

**Colon:** Strong vascular CD34 staining with no background staining.

# Figure 11

**Colon ERK Thr202/Tyr204:** Enhanced intensity of glandular epithelial staining in BHP compared to FFPE

**Colon ERK Thr202/Tyr204:** Enhanced intensity of staining in the carcinoma and transition areas in BHP compared to FFPE

**Breast ERK Thr202/Tyr204:** Enhanced intensity of staining in neoplastic ductal epithelium in BHP compared to FFPE.

**Breast GSK3αβ Ser21/Ser9:** Enhanced intensity of staining in neoplastic ductal epithelium in BHP compared to FFPE.

**Breast eIF4G Ser1108:** Enhanced intensity of staining in neoplastic ductal epithelium cytoplasm in BHP compared to FFPE.

**Mouse Brain p38 MAPK Thr180/Tyr182:** Cortex shows positive staining of occasional cell bodies in BHP with absence of staining in FF PE.

**Mouse Hippocampus Acetyl-CoA Carboxylase Ser79:** Positive staining of dendritic structures in BHP but negative staining in FFPE.

**B Human Colon ERK Thr202/Tyr204:** Retention of carcinoma cell staining after 3 months storage in BHP prior to paraffin embedding.

**Colon Cytokeratin 20:** Mucosa. Retention of carcinoma cell staining after 3 months storage in BHP prior to paraffin embedding.
